# Temporal Proteomic Characterization of SARS-CoV-2-Infected Mouse Lungs

**DOI:** 10.1016/j.mcpro.2026.101618

**Published:** 2026-07-02

**Authors:** Geun Hee Han, Misol Do, Dain On, Haengdueng Jeong, Jeong Jin Kim, Dohyun Han, Dongyoon Shin, Kyunggon Kim, Jung Seon Seo, Sung-Hee Kim, Jiseon Kim, Donghun Jeon, Hyuna Noh, Suhyeon Yoon, Sang Gyu Lee, Hyo-Jung Lee, Hong Bin Kim, Manki Song, Man-Seong Park, Kang-Seuk Choi, Jun Won Park, Jun-Won Yun, Jeon-Soo Shin, Ho-Young Lee, Hyuk Wan Ko, Jun-Young Seo, Ki Taek Nam, Daehee Hwang, Youngsoo Kim, Je Kyung Seong

**Affiliations:** 1 School of Biological Sciences, Seoul National University, Seoul, Republic of Korea; 2 Department of Biomedical Engineering, Seoul National University College of Medicine, Seoul, Republic of Korea; 3 Korea Mouse Phenotyping Center (KMPC), Seoul National University, Seoul, Republic of Korea; 4 Laboratory of Developmental Biology and Genomics, Research Institute for Veterinary Science, and BK 21 PLUS Program for Creative Veterinary Science Research, College of Veterinary Medicine, Seoul National University, Seoul, Republic of Korea; 5 Department of Molecular and Life Science, Hanyang University, Ansan, Republic of Korea; 6 Severance Biomedical Science Institute, Graduate School of Medical Science, Brain Korea 21 Project, Yonsei University College of Medicine, Seoul, Republic of Korea; 7 Biomedical Research Institute, Seoul National University Hospital, Seoul, Republic of Korea; 8 Department of Biomedical Sciences, School of Medicine, CHA University, CHA BIO COMPLEX, Gyeonggi-do, Republic of Korea; 9 Department of Medical Informatics and Biostatistics, University of Ulsan College of Medicine, Asan Medical Center, Seoul, Republic of Korea; 10 Interdisciplinary Program for Bioinformaics, Seoul National University, Seoul, Republic of Korea; 11 Department of Periodontology, Section of Dentistry, Seoul National University Bundang Hospital, Seongnam, Republic of Korea; 12 Department of Internal Medicine, Seoul National University Bundang Hospital, Seoul National University College of Medicine, Seongnam, Republic of Korea; 13 Science Unit, International Vaccine Institute, Seoul, Republic of Korea; 14 Department of Microbiology, Institute for Viral Diseases, Biosafety Center, Korea University College of Medicine, Seoul, Republic of Korea; 15 Laboratory of Avian Diseases, Research Institute for Veterinary Science, and BK 21 PLUS Program for Creative Veterinary Science Research, College of Veterinary Medicine, Seoul National University, Seoul, Republic of Korea; 16 Division of Biomedical Convergence, College of Biomedical Science, Kangwon National University, ChunCheon, Republic of Korea; 17 Laboratory of Veterinary Toxicology, College of Veterinary Medicine, Seoul National University, Seoul, Republic of Korea; 18 Institute of Immunology and Immunological Diseases, Yonsei University College of Medicine, Seoul, Republic of Korea; 19 Department of Microbiology, Yonsei University College of Medicine, Seoul, Republic of Korea; 20 Department of Nuclear Medicine, Seoul National University Bundang Hospital, Seongnam, Republic of Korea; 21 Department of Nuclear Medicine, Seoul National University, College of Medicine, Seoul, Republic of Korea; 22 Department of Biochemistry, College of Life Science & Biotechnology, Brain Korea 21 PLUS Program, Initiative for Biological Functions & Systems, Yonsei University, Seoul, Republic of Korea; 23 BIO MAX Institute, Seoul National University, Seoul, Republic of Korea

**Keywords:** SARS-CoV-2, K18-hACE2 mouse, proteomics, time-course analysis of lung proteome, nonsense-mediated decay, phagocytosis, neutrophil extracellular traps

## Abstract

Understanding dynamic activations of pathophysiological processes in the infected lungs is important for effective treatment of SARS-CoV-2 infection. Time-course transcriptome analyses of infected lungs have been performed to address this issue. Since proteins actually execute the pathophysiological processes, however, the time-course transcriptome data provide limited information regarding their temporal transitions. Here, we present time-course proteomic profiling of lung tissues from K18-hACE2 transgenic mice using liquid chromatography-tandem mass spectrometry analysis at day 0, 1, 2, 5, and 7 after SARS-CoV-2 infection. Clustering analysis to identify early, intermediate, and late up-regulated proteins, analysis of pathways enriched by these proteins, and network analysis of early, intermediate, and late upregulated pathways revealed detailed dynamic activations of molecular networks perturbed upon infection, and further proposed five markers that represented early-to-intermediate activation of nonsense-mediated decay (Smg6 and Upf1), intermediate-to-late activation of phagocytosis (Fcgr4 and Lamp2), and late activation of neutrophil extracellular trap formation (Padi4) that could be associated with severe pathological transitions. Immunohistochemistry analysis confirmed these temporal upregulation patterns of the five markers identified from the time-course proteome data, and immunofluorescence analysis further confirmed that Padi4 was up-regulated predominantly in neutrophils at the late stage. Our temporal proteomic analysis suggests potential pathways and molecule markers that can be used to predict severe pathological transitions during the course of SARS-CoV-2 infection.

## Highlights

• Time-course proteome profiles of mouse lungs during SARS-CoV-2 infection.
• Dynamic activation of cellular pathways during the course of SARS-CoV-2 infection.
• Pathways related to COVID-19 severity include NMD, phagocytosis, and NETosis.
• Representative proteins for these pathways can be used to predict COVID-19 severity.

## Graphical Abstract

**Figure:**
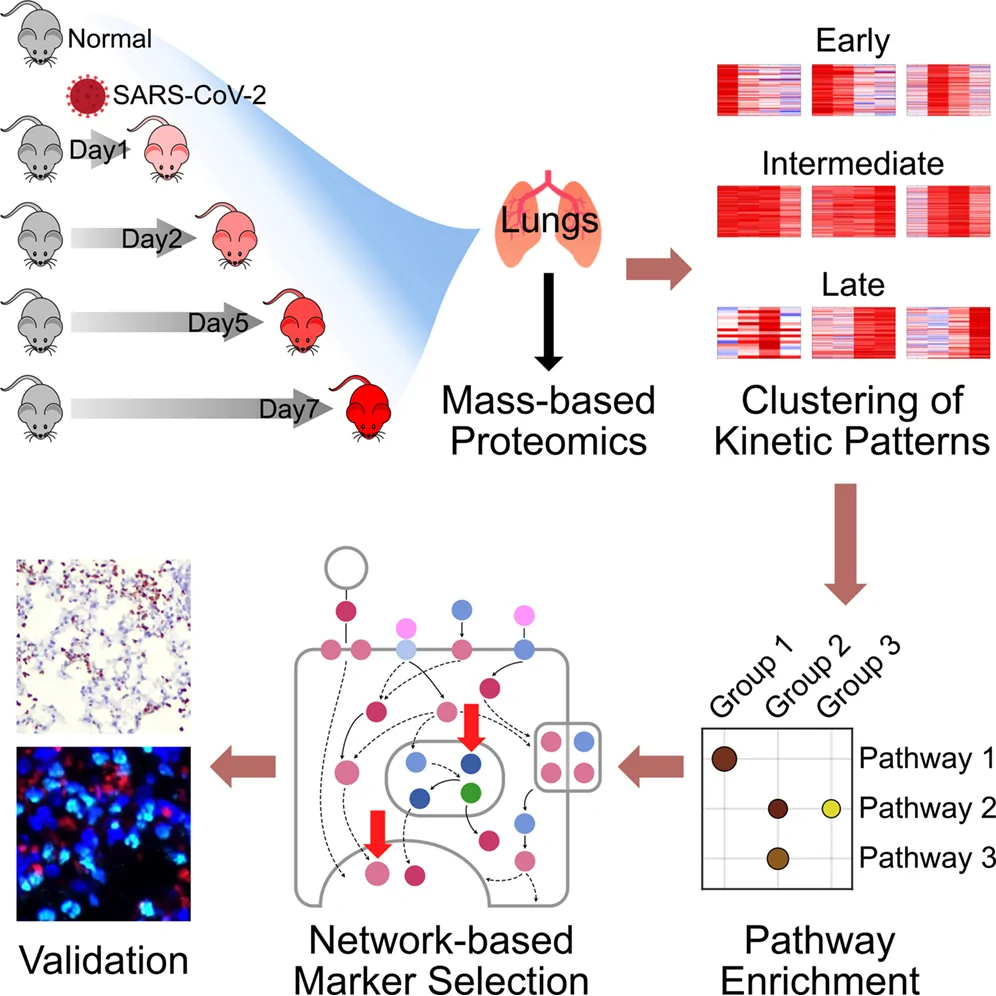

## In Brief

Decoding temporal transition of the proteome along SARS-CoV-2 infection is essential to understand sequential activations of key pathophysiological processes. We provide time-course proteome profiles of lungs at multiple post-infection days using K18-hACE2 mouse models. Integrative analyses of proteins changing over time revealed three pathways (nonsense-mediated decay, phagocytosis, and NETosis) and their representative biomarkers, which can be used to predict COVID-19 severity. Our data can serve as a comprehensive resource for many studies involving analyses of temporal proteome changes along SARS-CoV-2 infection.

Diverse pathophysiological processes are dynamically activated during the course of SARS-CoV-2 infection. Understanding of this dynamic pathophysiological activation program in the infected lungs is important for effective treatment of patients with SARS-CoV-2 infection because temporal transitions of activated processes are often associated with disease severity and prognosis. de Bruin *et al.* (1) studied prospectively 219 patients with COVID-19. Serial blood sampling was done to measure the levels of 64 biomarkers in cellular pathways related to COVID-19 pathophysiology. They found that the unfavorable outcome was associated with early immune processes (e.g., chemotaxis and interleukin production), while the death was associated with both early and late immune processes (e.g., chemotaxis, innate host response mechanisms, and wound healing). These results suggest that severity is closely linked to temporal activation of immune processes during the course of SARS-CoV-2 infection. However, understanding of specific pathways temporally activated along the infection and the molecules representing activation of these pathways at certain time points remains still limited.

To decipher molecular networks associated with COVID-19 pathophysiology, a number of transcriptomic and proteomic studies have been carried out for lung tissues from autopsies of COVID-19 patients. For example, Wu *et al.* (2) profiled transcriptomes of autopsy lung tissues from nine patients and control lung tissues from 10 cancer patients and identified 1470 up-regulated genes in COVID-19 samples, which are associated with neutrophil activation and extracellular structure organization linked to neutrophil extracellular trap formation (NETosis) and pulmonary fibrosis, respectively. Moreover, Nie *et al.* (3) generated proteome profiles of 30 autopsy lung tissues from 15 COVID-19 patients and seven control lung tissues from six victims of injury and identified 1606 up-regulated proteins in COVID-19 samples associated with cell proliferation, mitochondrial dysfunction, and immune response. Among these proteins, they proposed cathepsin L1 (CTSL1), an endosomal protease involved in viral entry, as a potential therapeutic target.

However, these mRNA or protein profiles provide snapshots of molecular changes in the infected lung tissues at certain time points after infection but lack information regarding transitions of perturbations in molecular networks associated with COVID-19 pathophysiology. Several time-course transcriptomic analyses of lung tissues have been reported. Winkler *et al.* (4) profiled transcriptomes of lung tissues obtained from SARS-CoV-2 infected K18-hACE2 transgenic mice at four time points (day 0, 2, 4, and 7) after infection and identified up-regulated genes in day 2, 4, or 7 post infection compared to day 0. The up-regulated genes at early stage (day 2 and 4) were highly shared, but a majority of them at late stage (day 7) were unique. Up-regulation of cytokine, type II IFN, and toll-like receptor signaling, as well as neutrophil activation, was most pronounced at late stage, and different sets of molecules in type I IFN signaling were up-regulated between early and late stages. Of note, proteins actually govern temporal transition of pathophysiological processes along SARS-CoV-2 infection, and the transcriptomic data thus provide only limited information of the temporal transition. However, no time-course proteomic profiling of SARS-CoV-2 infected lung tissues has been reported yet.

In this study, we present time-course proteomic analysis of lung tissues obtained from K18-hACE2 transgenic mice using liquid chromatography-tandem mass spectrometry (LC-MS/MS) analysis at five time points (day 0, 1, 2, 5, and 7) after SARS-CoV-2 infection. After identifying 1) early, intermediate, and late upregulated proteins after infection through clustering analysis and 2) cellular pathways enriched by these proteins, network analysis of the enriched pathways at early, intermediate, and late stages provided detailed temporal transitions of molecular networks activated along SARS-CoV-2 infection, and further identified key pathways for temporal transition between early, intermediate and late stages: (1) nonsense-mediated decay for early-to-intermediate transition; (2) phagocytosis for intermediate-to-late transition; and (3) NETosis for late stage activation. The representative markers for these pathways can be used to predict transitions to severe pathology during the course of SARS-CoV-2 infection.

## Experimental Procedures

### Cells and Viruses

Vero E6 (ATCC, CRL-1586) cells were cultured and maintained in Dulbecco’s Modified Eagle’s Medium (DMEM) supplemented with 10% fetal bovine serum (FBS; Hyclone) and 1% antibiotics, penicillin-streptomycin (Cytiva) (5). SARS-CoV-2 (NCCP43326, BetaCoV/Korea/KCDC03/2020) was obtained from the National Culture Collection for Pathogens, Korea Disease Control and Prevention Agency (6) and amplified in Vero E6 cells.

### Virus Propagation

Vero E6 cells were inoculated with SARS-CoV-2 isolate at one multiplicity of infection (MOI), and fresh DMEM with 2% FBS and 1% antibiotics was added. The cytopathic effects consisting of rounding and detachment of cells were observed in the whole area of the flask at 48 h after infection. The supernatants of virus-infected cells were harvested and stored at −80 °C. Viral titers were determined by plaque assay on Vero E6 cells as described previously (7, 8). All work with SARS-CoV-2 was conducted in biosafety level 3 facilities in accordance with the guidelines of and approved by the Institutional Biosafety Committee of Yonsei University Health System.

### Mice and *In Vivo* Experiment

K18*-*hACE2 transgenic mice under the *C57BL*/*6J* background were purchased from Jackson Laboratories. All animal experiments were conducted in a biosafety level 3 (BSL3) facility in accordance with the Public Health Service Policy on Humane Care and Use of Laboratory Animals. The protocol was approved by the IACUC of the Department of Laboratory Animal Resources of Yonsei University College of Medicine accredited by AAALAC International (#001071). To establish SARS-CoV-2 infection in the K18-hACE2 mice, 12-week-old male mice were anaesthetized using a zoletil-rompun mixture (4:1) and intranasally inoculated with 1 × 10^6^ plaque-forming units (PFU) of SARS-CoV-2. The body temperature and weight were measured daily until 7 days post infection (dpi) using an electronic scale and an implantable programmable temperature transponder (BMDS, DE, USA). For animal welfare, mice with body temperature under 25 °C were considered dead. Of note, we used no balanced set of male and female mice based on the previous report (9) that K18-hACE2 mouse model showed no significant gender sensitivity with similar clinical outcomes and infection sites in both male and female mice. To avoid the pre-maturation effect and senescence effect on our proteomics data, we used mature adult mice (12-week-old) in all experiments.

### Plaque Assay

Vero E6 cells were seeded into 12-well plates at 24 h before infection. Cells were inoculated with ten-fold serial dilutions of SARS-CoV-2 samples and adsorbed at 37 °C in a 5% CO2 incubator for 1h, rocking every 15 min. After the adsorption, the virus inoculum was removed from cells, and then 1.5 ml of 1% agarose (Lonza 50101) prepared in DMEM with 2% FBS and 1% antibiotics was added. The cells were incubated at 37 °C for 48 to 72 h. To stain plaques performed with an agarose overlay, 4% paraformaldehyde (Biosesang, Gyeonggi-do) was added on top of the agarose and incubated for 24 h at 4 °C. The agarose plug was removed, and the fixed monolayer was stained with 0.5% crystal violet in 20% methanol. The monolayers were washed with tap water. The number of plaques was counted. Viral titers were calculated in PFU per milliliter. For mice infected with SARS-CoV-2, tissues were isolated and chopped in Phosphate Buffered Saline (PBS) and stored at −80 °C until further processing. Viruses were extracted from the chopped tissues by three freeze-thawing cycles. The virus supernatant was collected and clarified by centrifugation and 0.2 μm filter. Virus titers were determined using the plaque assay as described above.

### Sample Preparation for Proteomic Analysis

Twenty-four mouse lung tissue samples were resected and subjected to the previously described sample preparation method with some modifications (10, 11, 12, 13). Tissue samples were first washed 6 times with sterilized PBS for 10 min and then incubated with sterilized PBS for 16 h at 4 °C. Washed tissues were homogenized and sonicated for 60 s (Sonics & Materials) in 300 μl lysis buffer (4% SDS, 2 mM TCEP in 0.1 M TEAB pH 8.5). After the samples were incubated for 20 min at 100°C, they were centrifuged to separate the supernatant from debris and other solid contents, and the pellet was discarded. The supernatant was transferred to the 0.22 μm pore costar filter (Corning Inc.) and centrifuged to obtain the filtered supernatant. Protein concentration of the sample was measured by bicinchoninic acid (BCA) reducing agent compatibility assay kit (Thermo Scientific). On the other hand, a pooled protein sample was prepared by taking 36 μg aliquots from all samples, respectively.

Protein digestion was performed by a combined approach including acetone precipitation and the filter-aided sample preparation (FASP) procedure described previously (10, 11, 12). Prior to the digestion step, the pooled protein sample and 150 μg of each tissue sample were precipitated with cold acetone (Sigma-Aldrich) at a ratio of 1:5 (sample: acetone, v/v). The mixture was vortexed thoroughly and then incubated overnight at −20 °C. The precipitate was centrifuged for 10 min at 15,000 rpm at 4 °C, and the acetone was gently removed. After an additional rinse step with 500 μl cold acetone and centrifugation, the protein pellet was air-dried for 2 h and stored for digestion. The acetone-precipitated samples (1 pooled tissue sample and 24 individual tissue samples) were mixed with 30 μl denaturation buffer (4% SDS, 0.1 M DTT, 0.1 M TEAB, pH 8.5). The mixture was vortexed gently and then boiled for 30 min at 95°C to denature the proteins. The denatured proteins were mixed with 300 μl 0.22-μm-pore filtered UA buffer (8 M urea, 0.1 M TEAB, pH 8.5) and then transferred to a 30-kDa centrifugal filter (Millipore). The resulting sample was centrifuged 3 times (14,000*g*, 15 min, 20 °C) to remove SDS. The washed samples were incubated in 200 μl 50 mM iodoacetamide (IAA) in UA buffer at room temperature (RT) for 30 min to alkylate the reduced cysteine. After exchanging the UA buffer with 40 mM TEAB, the samples were digested with 0.1 μg/μl trypsin/Lys-C at a ratio of 1:100 (enzyme: substrate, wt/wt) for 18 h at 37 °C. The tissue digests were measured by tryptophan fluorescence assay at 350 nm using an excitation wavelength of 295 nm to estimate the amounts of peptides (14).

### Tandem Mass Tags (TMT) Labeling

Because the number of samples exceeded the number of channels, we decided to distribute the 24 samples to three TMT experimental sets. All the samples were randomly allocated to the channels for each TMT set to have eight individual samples and two common pooled samples (Supplemental Fig. S1). Prior to the labeling step, 40 mM TEAB buffer was added to each 15 μg peptide sample to equalize the volume. A uniform volume of ovalbumin was then added to the samples. TMT reagents (0.8 mg) were dissolved in 110 μl of anhydrous ACN, of which 25 μl was added to identical channels in three experimental sets. ACN (174 μl) was then added to achieve a final concentration of 30%. The mixtures were incubated for 75 min at 23 °C. The reaction was quenched with hydroxylamine to a final concentration of 0.3% (v/v) for 15 min at 23 °C. The TMT-labeled samples per set were pooled and lyophilized to dryness in a speed-vacuum centrifuge.

### Sample Desalting and High-pH Reversed-Phase Peptide Fractionation

The TMT-labeled samples were re-dissolved in 0.1% trifluoroacetic acid (TFA) and desalted using HLB OASIS column according to the manufacturer’s instruction. Consequently, high-pH reversed-phase peptide fractionation was performed using Agilent 1260 bioinert HPLC (Agilent) equipped with a 300 Extended-C18 column (4.6 mm I.D × 25 cm long, 5 μm C18 particle). Peptide samples were separated with a 60 min linear gradient from 5% to 40% ACN in 15 mM ammonium hydroxide at a flow rate of 0.4 ml/min. The sample was fractionated into a total of 96 fractions, which were concatenated into 24 fractions with varying hydrophobicities to optimize coverage and LC-MS/MS run time, as previously described (15). The fractions were lyophilized in a vacuum centrifuge and stored at −80 °C until LC-MS/MS analysis.

### RP-Nano LC-ESI-MS/MS Analysis

The peptide samples were analyzed by a Q Exactive mass spectrometer equipped with an EASY-Spray ion source (Thermo Fisher Scientific), coupled to an Easy-nano LC 1000 (Thermo Fisher Scientific), following our established protocol (10, 11, 12). The peptides were separated on a 2-column setup that comprised a trap column (Thermo Fisher Scientific, 75 μm I.D. × 2 cm long, 3 μm Acclaim PepMap100 C18 beads, 100 Å) and an analytic column (Easy-Spray Pepmap RSLC, 75 μm I.D. × 50 cm long, 3 μm ReproSil-Pur-AQ C18 beads, 100 Å). Prior to sample injection, dried peptides were re-dissolved in solvent A (2% ACN and 0.1% Formic acid). The peptide samples were separated with an ACN gradient (6% to 90%) of 230 min by mixing solvent A (2% ACN and 0.1% v/v formic acid) and solvent B (100% acetonitrile and 0.1% v/v formic acid) in varying proportions in all samples, and data acquisition time was set to 210 min. Peptides that were eluted from the analytical column were ionized at a spray voltage of 2.2 kV in a positive ion mode. Mass spectra were collected in a data-dependent acquisition (DDA) manner using a top 15 method with 70,000 resolution at m/z 200 in a mass range of 350–1700 m/z. The automatic gain control (AGC) target value was 3 × 106 and the isolation window for MS/MS was 1.2 m/z. The 15 most abundant ions were fragmented by higher-energy collisional dissociation (HCD) with normalized collision energy (NCE) of 33 at a resolution of 35,000 at m/z 200. The AGC target value for MS/MS was 2 x 105. The maximum ion injection times for the survey and MS/MS scans were 30 and 120 ms, respectively. The dynamic exclusion time was set to 40 s to prevent repeated sequencing.

### Computational MS Data Analysis

The raw MS files were processed using Proteome Discoverer version 2.2 (Thermo Fisher Scientific). The tandem mass spectra search was conducted using the SEQUEST HT algorithm against the Uniprot *Mus musculus* database (December, 2015 released; 82,071 protein entries, http://www.uniprot.org) with the following parameters: full enzyme digest using trypsin (After KR/−) up to two missed cleavages; a precursor ion mass tolerance of 20 ppm (monoisotopic mass); a fragment ion mass tolerance of 0.02 Da (monoisotopic mass); static modifications of 229.163 Da on lysine residues and peptide N-termini for TMT labeling and 57.02 Da on cysteine residues for carbamidomethylation; and dynamic modifications of 42.01 Da for protein N-term acetylation and 15.99 Da for methionine oxidation. Using the established target-decoy search strategy (16), we identified proteins using the protein FDR <1%.

The proteins were then quantified using the “Reporter Ions Quantifier” node in Proteome Discoverer with co-isolation threshold = 70% and the default options for the other parameters. During this procedure, after adjusting the reporter ion intensities of the peptides by TMT deisotoping, the adjusted intensities for all peptides for each protein were summed to be the abundance of the protein. We then carried out the following normalization of these protein abundances for the subsequent analyses. For the identified 10,844 proteins, log_2_(protein abundances + 1) values were first calculated and normalized using the quantile normalization method (17) within each TMT sets. To obtain the relative abundances, the log_2_-fold-changes were computed by subtracting the median of the log_2_-intensities in the global intermediary samples within each corresponding TMT set from log_2_(protein abundances + 1) values. After concatenating all the normalized log_2_-fold-changes in 10 channels of each TMT set into a column vector, three column vectors of the concatenated log_2_-fold-changes for the three TMT sets were subjected to quantile normalization to remove the batch effects across TMT sets. The normalized log_2_-fold-changes were then reshaped into their original 10,844 × 24 matrix, where 24 is the number of lung samples excluding global intermediary samples. For each protein, we then auto-scaled its log_2_-fold-changes to have zero mean and unit standard deviation across the samples to prevent the bias toward the alteration patterns defined by the large log_2_-fold-changes and thus to select those with the alteration patterns defined by small log_2_-fold-changes. After the auto-scaling, the scale of auto-scaled log_2_-fold-changes between the samples became different. Subsequently, the *t*-statistic value for each time point versus non-infected control samples were calculated for orthogonal non-negative matrix factorization (ONMF) clustering (see below). However, the *t*-statistic value at each time point should be calculated at the common scale across samples. To match the scale across the samples, another quantile normalization was performed for the auto-scaled matrix. However, this can alter the distributional properties of the auto-scaled log_2_-fold-changes though not substantially. Such alterations did not corrupt the intention of the auto-scaling to prevent the bias toward the large log_2_-fold-changes.

### Clustering of Temporal Protein or mRNA Expression Profiles

For each protein, we obtained *t*-values from two sample *t* test to compare its log_2_-intensities in samples at each post-infection time point (day 1, 2, 5, or 7) with its baseline log_2_-fold-changes in samples at day 0. For the RNA-seq dataset (GSE154104) reported by Winkler *et al.* (4), we obtained the read count matrix from Gene Expression Omnibus. Fragments per kilobase of transcript per million mapped reads (FPKM) were calculated using the gene features (GTF file) of GRCm38.76. Log_2_(FPKM + 1) values were calculated and normalized using the quantile normalization method. Genes with log_2_(FPKM + 1) values available at all time points were used for further analysis. The same auto-scaling, quantile normalization, and *t*-value calculation used for the proteome data were applied to the normalized log_2_(FPKM + 1) values.

Finally, we applied ONMF clustering (18) to the normalized *t*-values of proteins or RNAs to identify clusters of proteins or RNAs based on expression patterns as previously described. The number of clusters (*k*) was set to 50. Among the resulting clusters, those with the up-regulation patterns were selected, and the clusters with similar alteration patterns were merged into groups.

### Identification of Molecular Signatures Defining the Clusters

The ONMF clustering method selects a set of the molecules (proteins or RNAs) belonging to cluster *i* as those with adjusted *p*-values ≤0.01 for the cluster (18). When multiple clusters with similar alteration patterns were merged into a group, the sets of molecules selected for the merged clusters were also combined to generate an initial set of molecules defining the group. This initial set of molecules can include false positives showing temporal expression patterns deviating from the representative pattern for the group. We next filtered out these false positives. To this end, for each group, we first selected the peak time point (e.g., day 1 for G1) with the highest mean *t*-value across the time points. For each molecule in the initial set, we then compared the log_2_-fold-changes (log_2_(FPKM + 1) for RNA data) in the samples at the peak time point with those in the samples at day 0 using a previously reported empirical two-sample *t* test and median-ratio test (19, 20). The final set of molecules defining each group was then selected as the molecules with *p*-value <0.1 (*t* test) and a median difference greater than 1.

### Functional Enrichment Analysis

To compute the enrichment *p*-value for each pathway (GOBP, Reactome, KEGG pathway, or WikiPathways), we employed an empirical hypergeometric testing approach based on random sampling from the reference (background) because Fisher’s exact test often results in *p* = 1 for many pathways when the group sizes are small. For each group (e.g., G8 including 171 proteins), we randomly sampled the same number of proteins (e.g., 171 proteins for G8) from the reference of 8277 proteins and counted the number of proteins involved in individual pathways. This random sampling was repeated 10,000 times. Using the resulting 10,000 counts for each pathway (e.g., NETosis), we estimated an empirical null hypothesis distribution of the count for the pathway and then a *p*-value as the number of random sampling trials producing the count larger than the observed count (e.g., seven for NETosis). Next, we computed FDRs (q values) for the *p*-values from the empirical testing using the Benjamini-Hochberg FDR correction as done in DAVID (alpha 0.05) (21). Of note, during the FDR correction, we removed the pathways with the non-meaningful observed counts smaller than the following cutoff: min(max(s, a),b), where s = ceil(5% of group size), a = 3, and b = 5. This cutoff means that the observed count should be larger than 5% of the group size, but at least 3 (a) and more, and also if the observed count is larger than 5 (b), we consider that such enrichment can be meaningful.

### Construction of Network Models

To construct a network model, we first selected proteins involved in the enriched GOBPs or pathways (e.g., “innate immune response”, “Nonsense-Mediated Decay”, or “phagocytosis”) (22), and Kyoto Encyclopedia of Genes and Genomes (KEGG) mouse pathways “Antigen processing and presentation”, “Complement and coagulation cascades”, “Fc gamma R-mediated phagocytosis”, “Phagosome”, and ‘Neutrophil extracellular trap formation” (23). We then constructed the network model showing interactions between the selected proteins, their interactors, and viral proteins based on the interactions in KEGG pathway database and the interactions reported in the previous literature (Supplemental Table S1).

### Immunostaining

For immunostaining of lung specimens, the SARS-CoV-2 infected tissues were fixed in 10% formalin for at least 24 h in biosafety level-3 condition. Afterwards, fixed tissues were embedded in paraffin, and 5-μm width of tissue sections were mounted in slides. The slides were immersed in xylene 3 times for 5 min and rehydrated in descending grade of alcohol (100%, 95% and 70%). After rehydration, slides were incubated in deionized water for 5 min, and antigen retrieval was performed using pH 6.0 citrate buffer for 15 min (Agilent). After antigen retrieval, slides were incubated in 3% hydrogen peroxide for 30 min to inhibit endogenous peroxidase and in protein blocking buffer (Agilent) for 1 h to inhibit non-specific signals. When murine derived antibodies were used, the mouse-on-mouse blocking solution (Vector laboratories) was additionally treated for 2 h. Afterwards, primary antibodies were treated in slides for 24 h at cold room. The following primary antibodies were commercially used: anti-SMG6 (Abcam, ab87539), anti-UPF1 (Abcam, ab109363), anti-CD16 (BD, 553142), anti-LAMP2 (Santacruz, sc-18822), anti-PADI4 (Abcam, ab96758), anti-LAMP3 (Novus, DDX0190P), anti-Ly6G/6C (Abcam, ab2557), anti-F4/80 (Abcam, ab6640). The sections were then incubated in horseradish peroxidase-conjugated secondary antibodies (Agilent) for 15 min. The immunohistochemistry signal was developed using DAB substrate (Agilent) and Mayer’s hematoxylin was used for nuclear staining. For immunofluorescence staining, primary antibodies were detected with Alexa 488-conjugated and Cy3-conjugated secondary antibodies. The immunofluorescence images were acquired using EVOS-FL and LSM 980 systems.

### Experimental Design and Statistical Rationale

Lungs were collected from five, six, six, five, and two mice at 0, 1, 2, 5, and 7 dpi, respectively as illustrated in Figure 1*A*, and, after protein isolation and digestion, these biological replicates were subjected to TMT labeling with no technical replicates as described in Supplemental Fig. S1. The number of replicates was determined to be five to six at each time point, which can provide statistical significance in identifying the differences of protein abundances between different time points according to a previous report (24). These replicate numbers are larger than three replicates commonly used in many omics studies for differential expression analysis using mouse samples (25). Of note, however, we could not but use only two mice at seven dpi because most of mice were dead at that time. Furthermore, to avoid the labeling-derived bias in the data at individual time points, we mixed up the replicates at each time point across different TMT sets (Supplemental Fig. S1). Also, we included the pooled mixture of the peptides from all samples as a universal control to remove the unwanted variation during peptide quantification between different TMT sets. The replicate number and TMT-labeling scheme are expected to collectively provide unbiased reliable statistical significance with reduced corruption of unwanted variation in the following data analyses.

**Fig. 1:**
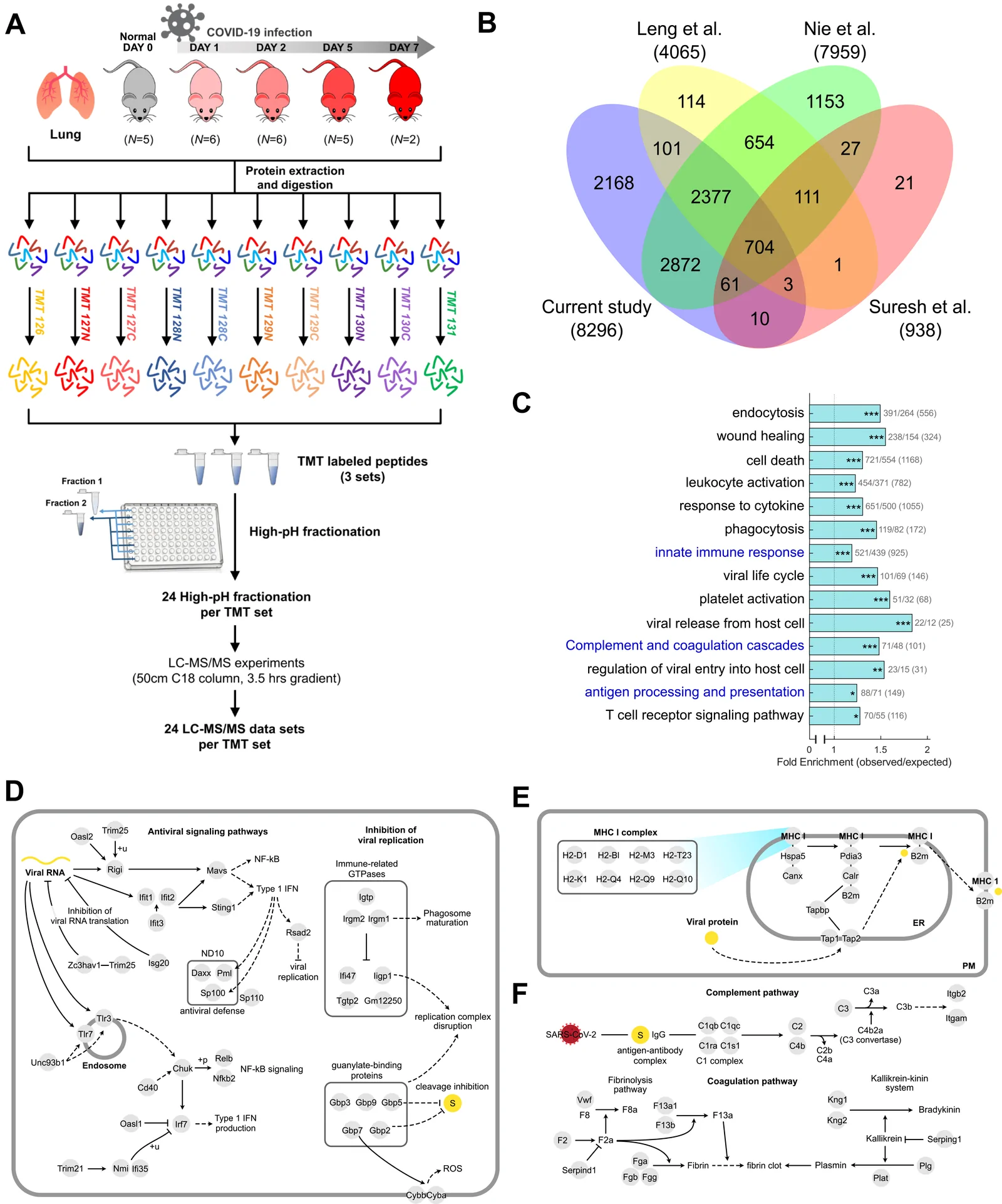
**Comprehensive proteome landscape of SARS-CoV-2 infected mouse lungs.***A*, overall scheme describing sample preparation, proteome profiling. See text and Methods for details. *B*, comparison of our lung proteome with three previously reported proteomes associated with SARS-CoV-2-infected lungs. The numbers of genes encoding the detected proteins in the four studies (parenthesis) and their overlaps are shown. *C*, GOBPs or pathways represented by the genes encoding the detected proteins. Each bar represents the fold enrichment defined by the ratio of the numbers of observed and expected genes annotated with the corresponding GOBP/pathway among the genes encoding the 10,844 detected proteins. Of note, the number of expected genes was estimated based on random chance, given the total number of genes annotated with the GOBP/pathway in the whole genome (reference). The ratios, as well as the total number of genes in parenthesis, are shown next to the bars for the indicated GOBP/pathways. ∗, q < 0.05; ∗∗, q < 0.01; ∗∗∗, q < 0.0001 by an empirical hypergeometric testing described in EXPERIMENTAL PROCEDURES. *D–F*, network models describing interactions among the experimentally identified and quantified proteins involved in the processes related to innate immune response (*D*), antigen processing and presentation (*E*), complement and coagulation cascades (*F*). Gray and yellow nodes indicate host and viral proteins, respectively. Solid and dashed edges represent direct and indirect activation (*arrow*) or suppression (bar-ended), respectively. Thick gray lines denote the plasma and nuclear membranes, and membranes of other organelles including endoplasmic reticulum (ER) and endosome.

## Results

### Comprehensive Proteome Landscape of SARS-CoV-2 Infected Mouse Lungs

For time-course proteomic analysis, we first collected lung tissues from the mock-infected and the SARS-CoV-2 infected mice at five time points (day 0, 1, 2, 5, and 7) after infection as shown in Figure 1*A*. We then performed 10-plex TMT labeling of the peptides resulted from FASP digestion of individual samples, which generated three sets of TMT-labeled peptide samples, as illustrated in Supplemental Fig. S1. For each TMT set, the peptide samples were desalted and fractionated into 24 fractions using high pH fractionation as previously described (15). Each fraction was then analyzed by LC-MS/MS using a Q Exactive mass spectrometer, resulting in 24 LC-MS/MS data sets. For the resulting 72 LC-MS/MS data (24 × 3 TMT sets), we performed peptide identification and protein quantification at each time point using Proteome Discoverer with the SEQUEST-HT algorithm and identified a total of 152,182 peptides (Supplemental Table S2) and 10,844 protein groups (Supplemental Table S3) which had at least one unique peptide with high confidence (FDR <1%).

To evaluate the comprehensiveness of our proteome, we next compared the 10,844 proteins with three previously reported proteomes of SARS-CoV-2 infected lung tissues generated using LC-MS/MS analysis. Leng *et al.* (26) identified 4128 proteins from lung tissues of deceased COVID-19 patients and control lung tissues of cancer patients; Nie *et al.* (3) identified 8013 proteins from lung tissues of deceased COVID-19 patients and non-infected controls; and Suresh *et al.* (27) identified 1585 proteins from lung tissues of SARS-CoV-2 infected and mock-infected Syrian golden hamsters (*Mesocricetus auratus*). To remove the bias from different protein isoform groups used, we mapped the identified proteins to human Entrez gene IDs based on the human–mouse and –hamster orthologue models and compared the mapped 8296, 4065, 7959, and 938 genes that encode 10844, 4128, 8013, and 1585 proteins, respectively (Fig. 1*B*). Our proteome included 78.4% (3185 out of 4065), 75.6% (6014 out of 7959), 82.9% (778 out of 938) of the proteomes detected from Leng *et al.*, Nie *et al.*, and Suresh *et al.*, respectively, and had 2168 protein-encoding genes uniquely identified, suggesting high comprehensiveness of our temporal proteome of SARS-CoV-2 infected lung tissues.

Given the comprehensiveness of our proteome, we next asked whether our proteome sufficiently covers cellular processes that have been shown to be involved in COVID-19 pathophysiology. To answer this question, we examined cellular processes (GOBP, Reactome, KEGG pathway, or WikiPathways) represented by the 10,844 mouse proteins by performing an empirical hypergeometric test based on random sampling. The enriched cellular processes/pathways included a spectrum of processes related to SARS-CoV-2 infection, such as cell death, innate immune response, viral life cycle, complement and coagulation cascades, phagocytosis, response to cytokine, and antigen processing and presentation (Fig. 1*C*).

To understand cellular networks associated with these processes, we built network models describing interactions among the proteins involved in three key processes (blue labeled in Fig. 1*C*)- innate immune response, antigen processing and presentation, and complement and coagulation cascades. The first network model (Fig. 1*D*) for the innate immune response shows antiviral signaling pathways involving: 1) inhibitors of viral RNA translation (Zc3hav1, Trim25, and Isg20); 2) regulators (Mavs and Sting1) of antiviral type I interferon signaling activated by viral RNA-recognizing sensors, Rigi and Ifit1/2/3, together with co-activators of Rigi (Oasl2 and Trim25); 3) regulators of Nfkb signaling (Nfkb2 and Relb) and antiviral Irf7 signaling activated by viral RNA receptors, Tlr3/7, via Cd40 and Chuk; and 4) ND10 complex proteins (Daxx, Pml, and Sp100) activated by antiviral type I interferon signaling and involved in antiviral defense and its associate protein Sp110. The network model also shows the pathways related to inhibition of viral replication including 1) GTPases (Ifi47, Iigp1, Tgtp2, and Gm12250), guanylate-binding proteins (Gbp2/3/5/7/9), and Rsad2 involved in disruption of viral replication; and 2) regulator GTPases (Irgm1/2 and Igtp) involved in phagosome maturation and modulation of Ifi47, Iigp1, Tgtp2, and Gm12250. See Supplemental Table S1 for references for the interactions shown in Figure 1*D*.

The second network model (Fig. 1*E*) shows the antigen processing and presentation pathways involving (1) transporters (Tap1/2 and Tapbp) of viral proteins into endoplasmic reticulum (ER); (2) proteins assisting the assembly, folding, and ER-to-plasma membrane (PM) trafficking of major histocompatibility complex (MHC) I (Hspa5, Canx, Pdia3, Calr, and B2m); and (3) MHC I proteins (H2-Bl/D1/K1/M3/Q4/Q9/Q10/T23). The third network model (Fig. 1*F*) shows the complement and coagulation pathways including (1) complement proteins (C1qb/1qc/1ra/1s1/2/3/4b) and receptor of C3b (Itgb2 and Itgam); (2) regulators of kallikrein-kinin system (Kng1/2) and fibrinolysis pathways [plasminogen (Plg), its activation (Plat), coagulation factors (F2, Vwf, and F13a1/b), and fibrinogens (Fga/b/g)] in coagulation system. These pathways enriched by the detected proteome and the network models collectively indicate that our proteome serves as a comprehensive resource for investigating dynamic activation of key cellular processes in the infected lungs during the course of SARS-CoV-2 infection.

### Temporal Transitions of Cellular Networks Activated along SARS-CoV-2 Infection

Before systematically identifying proteins with diverse upregulation patterns, we first examined whether our temporal proteome reflects known activation kinetics of the above key cellular processes-innate immune response (antiviral signaling and viral replication inhibition), antigen processing/presentation, and complement/coagulation. Most of the proteins involved in innate immune response (Fig. 1*D*) were up-regulated beginning day 1 (Fig. 2*A*, left). While some were up-regulated from day 1, a majority of proteins involved in antigen processing/presentation (Fig. 1*E*) were up-regulated beginning day 2 (Fig. 2*A*, upper right). Interestingly, the proteins involved in complement/coagulation (Fig. 1*F*) showed a mixed pattern of up-regulation at day 2 (e.g., Plat, C1qb, C4b, Sperping1, and C1ra) and at day 5/7 (e.g., Kng1/2, Itgb2/am, Serpind1, F2, Vwf, and F13a1) (Fig. 2*A*, lower right), which may reflect early activation of coagulation/complement at the local infection site and their late systematic activation in circulation. These activation kinetic patterns are consistent with those of the key processes previously reported (28, 29, 30).

**Fig. 2:**
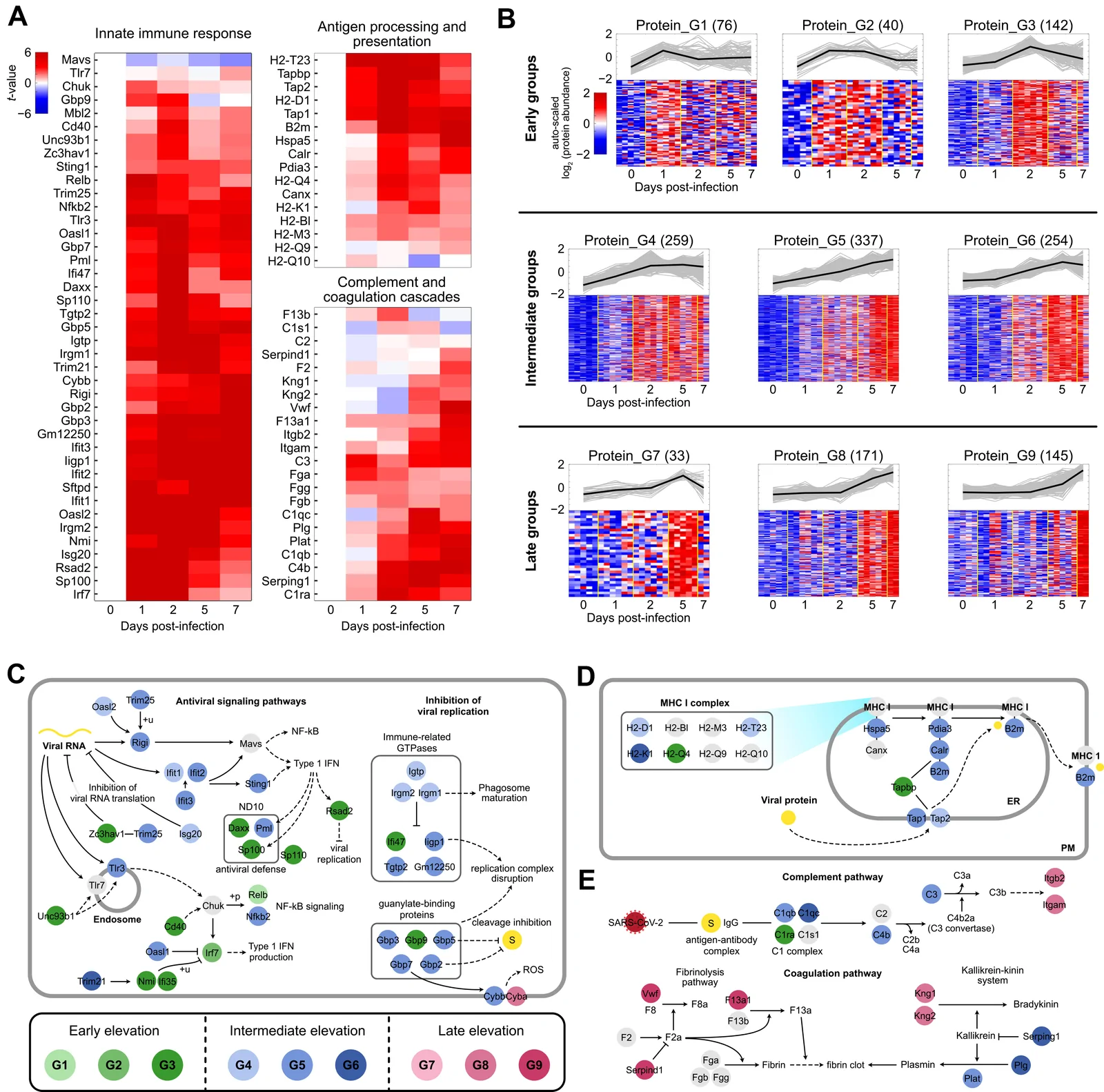
**Temporal transitions of cellular networks activated along SARS-CoV-2 infection.***A*, heat maps showing expression patterns of known key processes related to SARS-CoV-2 infection. Colors represent up-regulation (*red*) and down-regulation (*blue*) at day 1, 2, 5, and 7 compared to the control (day 0) after SARS-CoV-2 infection. The color bar denotes the gradient of *t*-values between day 0 and day 1, 2, 5, or 7 (e.g., day 1 vs. day 0 or day 2 vs. day0). *B*, heat maps showing up- or down-regulation patterns of proteins in G1–9 over time. The number of proteins in each group is denoted in parentheses. The color bar denotes the gradient of the auto-scaled log_2_(protein abundances) for individual proteins (*rows*). Profiles of individual proteins (*gray lines*) in each pattern are shown on top of the heat map together with the median profile (*black line*). *C–E*, early (*green*), intermediate (*blue*), or late (*magenta*) up-regulated proteins in the network models shown in Figure 1, *D*–*F*.

This consistency in the activation kinetics prompted us to systematically identify proteins elevated at early, intermediate, and late stages after SARS-CoV-2 infection by performing clustering analysis of our temporal proteome profiles. To reduce the bias from missing values, among the 10,844 proteins, we selected 8277 proteins that were detected in all three TMT sets (Supplemental Fig. S2, *A*–*C*) and applied ONMF clustering to these selected proteins, resulting in 16 up-regulated and 18 down-regulated clusters and 9 clusters with mixed patterns (Supplemental Fig. S2*D*). We focused on 21 clusters showing early, intermediate, and late up-regulation and merged them into three early (G1-3 in Fig. 2*B*), three intermediate (G4-6), and three late groups (G7-9) of the proteins based on their up-regulation patterns, as illustrated in Supplemental Fig. S2*E* (Supplemental Table S3, ‘Groups’). The early groups showed transient elevation at day 1 (G1), day 2 (G3), or both days (G2). In contrast, the intermediate groups showed sustained elevation beginning day 1 (G4 and 5) or day 2 (G6). The late groups showed transient elevation at day 5 (G7) or prolonged elevation from day 5 to 7 (G8 and 9). These different elevation patterns suggest possible programs underlying the upregulation kinetics duration during the course of SARS-CoV-2 infection.

We next attempted to examine whether these temporal upregulation patterns identified from mouse SARS-CoV-2 infected lungs were consistent with those in human SARS-CoV-2 infected lungs. However, we found no time-course proteomic analysis of lungs from COVID-19 patients because it is almost impractical to have multiple biopsies of the same patient’s lung during the course of infection. On the other hand, there have been several static proteomic analyses of autopsy lung tissues from patients (3, 26, 31, 32). We could not thus compare our temporal (early, intermediate, and late) changes with those in these static datasets but analyze whether the temporal changes were observed consistently in the previous static proteome data. For this analysis, among the static proteome datasets, we chose the largest lung proteome dataset generated from (1) 30 autopsy lung tissues from 15 deceased patients with COVID-19 and 2) seven control lung tissues from six victims of injury (3). We first obtained the list of differentially expressed proteins (DEPs; 1259 up-regulated and 347 downregulated) between the COVID-19 autopsy and control samples and then identified the overlapping DEPs with those in our G1-9 in Figure 2*B*. The comparison revealed significant (adjusted *p* < 0.05) overlaps of the up-regulated proteins with those in G4-9 (intermediate and late), but no significant (adjusted *p* > 0.30) overlaps of the down-regulated proteins (Supplemental Fig. S2*F*). According to the WHO severity definitions (33) [‘control’ (non-infected), ‘non-severe’ (non-hospitalized), ‘severe’ (hospitalized), and ‘critical’ (ventilated or death)], all these autopsy samples belonged to ‘critical’ and could be thus considered close to our late stage from day 5 when mice began to die. The significant overlaps of the up-regulated proteins in the autopsy samples with G4-9 showing the up-regulation at late stage further support the validity of our temporal up-regulation patterns.

We further mapped these early, intermediate, and late up-regulated proteins into the network models in Figure 1, *D*–*F*. The proteins in the innate immune response network were clustered to either early or intermediate group (Fig. 2*C*). Particularly, the proteins in ND10 complex involved in antiviral defense (Daxx and Sp100) and the antiviral Irf7 signaling (Cd40, Irf7, Nmi, and Ifi35) showed early elevation (green nodes in Fig. 2*D*) while those in the other pathways showed mostly intermediate elevation (blue nodes in Fig. 2*D*). Most of proteins in the antigen processing/presentation network showed intermediate elevation. In the complement/coagulation network, many molecules, including 1) the C3b receptors (Itgb2/am), 2) the proteins (Vwf, Serpind1, and F13a1) in fibrinolysis pathway, and 3) precursors of bradykinin (Kng1/2) showed late elevation (magenta nodes in Fig. 2*E*) while others, including the complement proteins (C1qb/1qc/4b/3) and plasmin metabolism in the kallikrein-kinin system (Plg, Plat, and Serping1) showed intermediate elevation. Taken together, these results suggest that our temporal proteome can be useful to decipher dynamic activation of cellular networks along SARS-CoV-2 infection.

### Cellular Processes Associated with Pathological Transitions

Understanding temporal transitions of pathophysiological processes during the course of SARS-CoV-2 infection is important because some of these transitions are linked to transition to severe pathology. To examine temporal activations of cellular pathways, we performed enrichment analyses of the proteins in G1-9 using an empirical hypergeometric testing method described in experimental procedures and estimated the activation strength of each pathway at early, intermediate, and late stages as *Z*-scores of the enrichment significance (q-value) (Fig. 3*A*). Consistent with the findings in Figure 2, *C*–*E*, innate immune response and antigen processing/presentation began to be activated from early stage and showed sustained activation at the intermediate stage, (green labeled pathways in Fig. 3*A*). Also, the complement activation and coagulation pathways began to be activated from intermediate stage and showed sustained activation at late stage (magenta-labeled pathways in Fig. 3*A*).

**Fig. 3:**
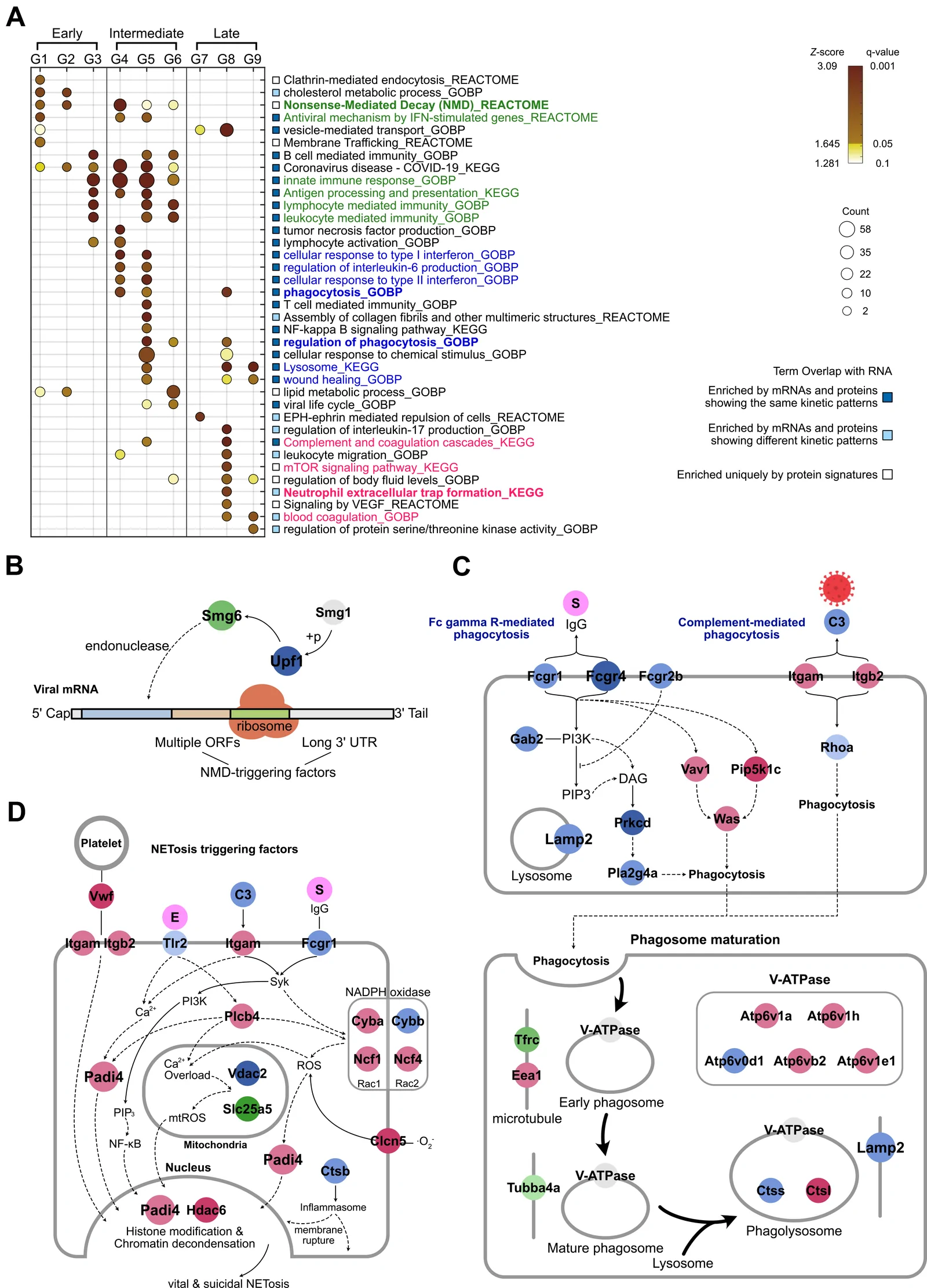
**Cellular processes associated with pathological transitions.***A*, GOBPs or pathways represented by the proteins in G1–9 (q-value <0.05). Dot color denotes *Z*-scores computed as −N^−1^(q), where q is the enrichment q-value of the pathway computed by an empirical hypergeometric testing described in experimental procedures using the reference (background) of 8277 proteins used for NMF clustering, and N^−1^ is the inverse normal distribution. Dot size denotes the number of proteins involved in the corresponding GOBPs or pathways. Colored boxes next to each term indicate whether the term is also enriched by the up-regulated mRNAs (RNA_G1–5 in Supplemental Fig. S4*A*) with the same (*dark blue*) or different (*light blue*) kinetic patterns. White boxes indicate no enrichment by the up-regulated RNA patterns. Label colors represent GOBPs or pathways showing early-to-intermediate transition (*green*), intermediate activation (*blue*), and late activation (*magenta*). *B–C*, network models describing interactions among the up-regulated proteins involved in GOBPs or pathways related to NMD (*B*), phagocytosis-related processes (*C*), and NETosis (*D*). Node colors represent early (*green*), intermediate (*blue*), or late (*purple*) upregulated proteins. See the legend of Figure 2, *C*–*E* for node colors and the legend of Figure 1, *D*–*F* for edges and membranes. Proteins selected for further experimental validation were indicated by larger node sizes.

Interestingly, there were cellular pathways that were activated from early stage and prolonged to intermediate stage, including nonsense-mediated decay (NMD), antiviral mechanism by IFN-stimulated genes, lymphocyte or leukocyte mediated immunity (green labeled pathways in Fig. 3*A*). These pathways may play important roles in transition from early to intermediate pathology. Of them, NMD pathway is known to be an important defense mechanism against *viruses* including SARS-CoV-2 (34) and was thus reported to be a key process targeted by SARS-CoV-2 at early stage of infection (35). The network model (Fig. 3*B*) further showed the early up-regulated Smg6 and the intermediate up-regulated Upf1, central factors in NMD pathway. Taken together, these data suggest that the NMD pathway can be central to the early-to-intermediate transition, which may serve as an early determinant of disease severity, and up-regulation of Smg6 and Upf1 can be used to predict the severity at early and intermediate stages.

There were also cellular pathways that were activated from the intermediate stage and prolonged to the late stage, including phagocytosis, lysosome, and wound healing (blue labeled pathways in Fig. 3*A*). Of them, upregulation of phagocytosis at the intermediate stage can provide the increased flux of viral antigen into the antigen processing/presentation pathway activated from early stage, thereby facilitating transition to adaptive immunity. The network model (Fig. 3*C*) showed the proteins involved in 1) Fc gamma receptor- and complement-mediated phagocytosis and 2) phagosome maturation with mixed patterns of intermediate and late up-regulation. For example, in the Fc gamma receptor-mediated phagocytosis, Fc gamma receptors (Fcgr1/4/2b) and their downstream factors (Gab2, Prkcd, and Pla2g4a) showed intermediate elevation while other downstream factors (Vav1, Pip5k1c, and Was) showed late elevation. These data suggest that the representative proteins for phagocytosis (e.g., Fcgr4 and Lamp2, large nodes in Fig. 3*C*) can be used to predict severity at intermediate and late stages.

There are cellular pathways predominantly activated at late stage, including complement/coagulation, mTOR signaling, and NETosis, which can directly contribute to severity. Of them, NETosis is commonly observed in severe COVID-19 patients (36, 37, 38). The network model shows that NETosis-triggering factors were up-regulated at the intermediate stage (Vwf and Itgam/b2), and late stage (Tlr2, C3, Fcgr1, and Clcn5) (Fig. 3*D*, top). Moreover, the downstream pathways of these factors were also mostly elevated at late stage, including NADPH oxidases (Cyba and Ncf1/4), phospholipase C beta 4 (Plcb4), and chromatin decondensing enzymes (Padi4 and Hdac6) (Fig. 3*D*, middle and bottom) while some downstream pathways molecules were found elevated at intermediate stage, including NADPH oxidase component (Cybb), mitochondrial channel protein (Vdac2), and cathepsin B (Ctsb). The mixed elevation patterns indicated that NETosis is initiated at the intermediate stage and manifested at the late stage, suggesting that elevation of the representative NETosis factors at the late stage can be used to predict severity at late stages of SARS-CoV-2 infection.

To examine whether the above three pathways (NMD, phagocytosis, and NETosis) are consistently up-regulated in human SARS-CoV-2 infected lungs, we next identified cellular pathways represented by the overlapping DEPs from the aforementioned autopsy data with those in G4-9 using DAVID. The overlapping DEPs significantly represented (1) NMD, (2) regulation of phagocytosis, and (3) neutrophil degranulation and extracellular trap formation (Supplemental Fig. S3*A*), as well as other pathways related to those activated at early, intermediate, late stages in Figure 3*A*. Moreover, several studies have profiled proteomes of serum samples from control, non-severe, severe, and critical cases (39, 40, 41, 42). We thus examined how the three pathways were associated with the severity definitions in the serum proteome data. To this end, among several datasets, we chose one of the largest serum proteome datasets, Olink proteome data generated from serum samples of 631 patients (182 controls, 181 non-severe, 135 severe, and 133 critical cases). After NMF clustering, we identified three severity-associated up-regulation patterns and further cellular pathways represented by the proteins in the three patterns (Supplemental Fig. S3, *B*–*D*). Regulation of phagocytosis and neutrophil degranulation/migration related to NETosis were most significantly activated in severe and critical cases. Of note, however, the association of NMD pathway selected to represent early-to-intermediate transition could not be analyzed because the Olink panel included no proteins involved in NMD pathway. Taken together, these results suggest that many of the pathways represented by G4-9, including NMD, phagocytosis, and NETosis for pathophysiological transitions, were consistently up-regulated in the human proteome data, and also support the association of the three pathways with severity transitions.

### NMD, Phagocytosis, and NETosis as Indicators of Pathological Transitions

Before selecting proteomic signatures associated with the activation of NMD, phagocytosis, and NETosis pathways, we examined whether the temporal activation patterns of these pathways were also observed at the mRNA level. To this end, we obtained a previously reported mRNA-sequencing dataset (GSE154104) generated from mouse lung tissues at day 0, 2, 4, and 7 after SARS-CoV-2 infection (4). We then performed the same analyses to the time-course RNA data. The ONMF clustering analysis identified 19 clusters, which were merged into early (RNA_G1), intermediate (RNA_G2), and late clusters (RNA_G3-5) based on their up-regulation patterns (Supplemental Fig. S4*A* and Supplemental Table S4). Comparison of the kinetic patterns between mRNA and protein clusters revealed the shared kinetic patterns (Supplemental Fig. S4*B*, left): 1) early (Protein_G3-RNA_G1); 2) intermediate (Protein_G4-RNA_G2 and Protein_G5-RNA_G3); and 3) late up-regulation patterns (Protein_G7-RNA_G4 and Protein_G9-RNA_G5). However, protein data provided additional up-regulation patterns (early-Protein G1-2; intermediate-Protein G6; and late-Protein G8) (Supplemental Fig. S4*B*, right). Further comparison of mRNA and protein signatures showed that the overlaps between mRNAs and proteins were significant for the above pairs of the shared early and intermediate up-regulation patterns (blue boxes in Supplemental Fig. S4*C*). Interestingly, however, the shared late up-regulation pairs (Protein_G7-RNA_G4 and Protein_G9-RNA_G5) showed no significant overlaps.

We next performed functional enrichment analysis of genes in RNA_G1-5 (Supplemental Fig. S4*D*). For comparison of the pathways enriched by mRNA and protein signatures, we categorized the pathways into Class 1 enriched by mRNAs and proteins showing the same kinetic patterns; Class 2 enriched by mRNAs and proteins showing different kinetic patterns; Class 3 enriched uniquely by protein signatures; and Class 4 enriched uniquely by mRNA signatures (Supplemental Fig. S4*E*). Among the NMD, phagocytosis, and NETosis pathways, Class 1 included phagocytosis enriched by the shared intermediate mRNAs and proteins in Protein_G4-RNA_G2 and Protein_G5-RNA_G3 (Supplemental Fig. S4*F*, ‘Phagocytosis’). Class 2 included NETosis enriched by proteins in Protein_G8, a unique kinetic pattern in the protein data, and by mRNAs in the intermediate up-regulation pattern RNA_G3 (Supplemental Fig. S4*F*, ‘NETosis’). Class 3 included NMD enriched by proteins in the unique early up-regulation patterns Protein_G1 and G2. Interestingly, the genes encoding these NMD-related proteins were conversely down-regulated, suggesting differential regulation of NMD at mRNA and protein levels (Supplemental Fig. S4*F*, ‘NMD’). Considering the shared or distinctive nature of Classes 1 to 3 between mRNAs and proteins, protein signatures representing these three pathways provide complementary information to mRNA signatures, which can improve our understanding of severity-associated pathological transitions. Of note, Class 4 included the pathways enriched by mRNAs, but not by proteins in the Protein-RNA pairs of the shared up-regulation patterns. For example, some of proteins involved in PI3K-Akt signaling showed no significant up-regulation, differently from their mRNAs, suggesting their differential regulation at mRNA and protein levels (Supplemental Fig. S4*F*, ‘PI3K-Akt signaling pathway’).

To confirm the activation of NMD, phagocytosis, and NETosis pathways, we next performed immunohistochemistry (IHC) analysis of the following representative proteins for NMD, phagocytosis and NETosis pathways in mouse lung tissues at day 0, 2, 5, and 7 after SARS-CoV-2 infection: two central regulators Smg6 (early) and UPF1 (intermediate) in the NMD network (Fig. 3*B*), a key regulator in Fc gamma receptor-mediated phagocytosis, Fcgr4 (intermediate), and phagosome maturation, Lamp2 (intermediate) in the phagocytosis network (Fig. 3*C*), and a key histone citrullinating enzyme of reactive oxygen species (ROS) pathway, Padi4 (late), in the NETosis network (Fig. 3*D*). The IHC analysis revealed that Smg6 increased faster than Upf1 at day 2 (Fig. 4, *A*–*C*), consistent with its early up-regulation pattern identified from the proteome data. However, elevation of Upf1 at day 2 was still significant compared to day 0 (Fig. 4*C*), confirming its intermediate up-regulation in the proteome data. Phagocytosis markers Fcgr4 and Lamp2 showed gradual increases in expression beginning day 2 (Fig. 4, *A*, *D* and *E*), and NETosis marker Padi4 showed predominant increase at day 7 (Fig. 4, *A* and *F*), confirming their intermediate (Fcgr4 and Lamp2) and late (Padi4) up-regulation in the proteome data.

**Fig. 4:**
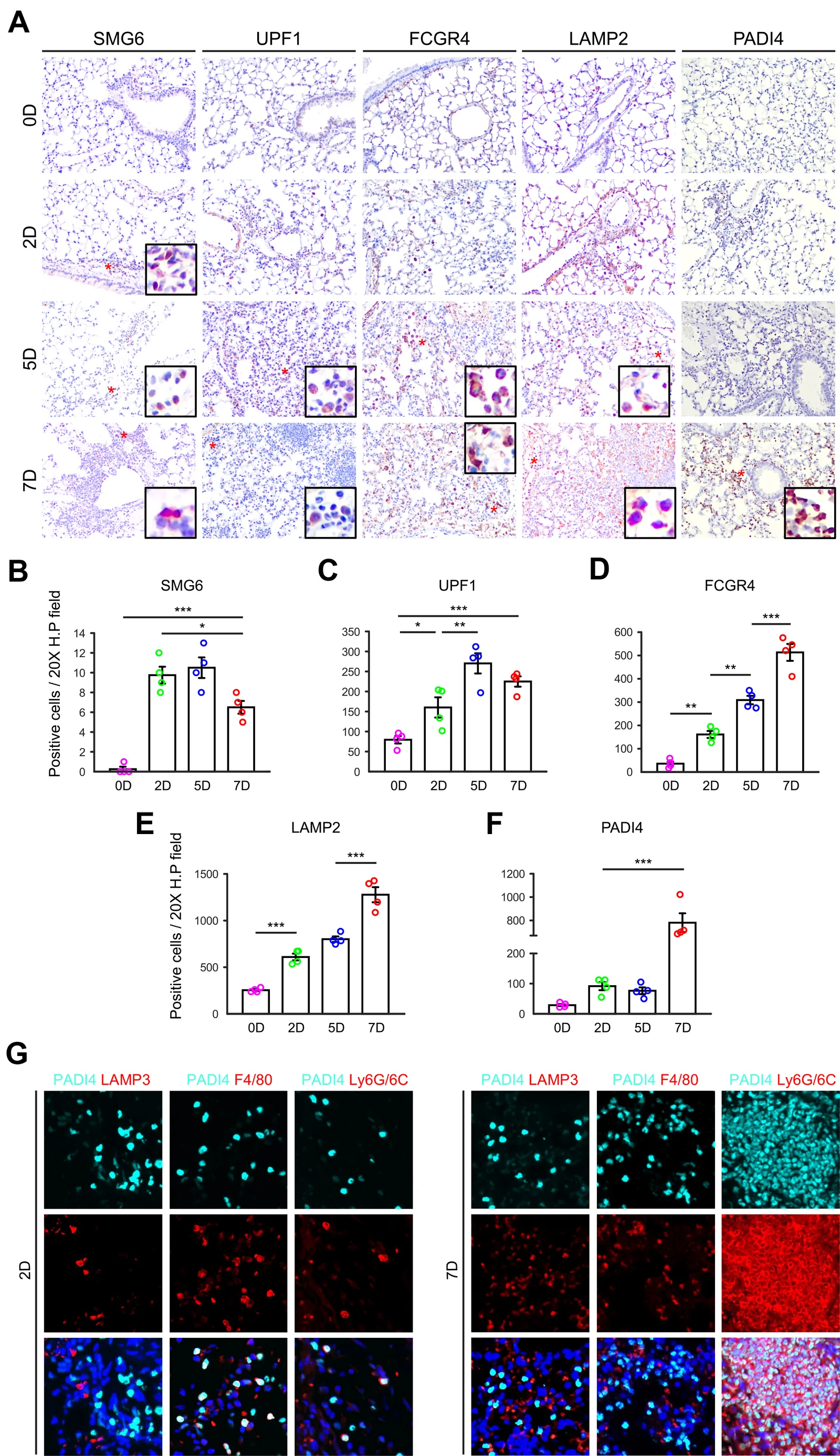
**Immunostaining of NMD, phagocytosis and NETosis markers.***A*, representative IHC images for NMD markers (Smg6 and Upf1), phagocytosis markers (Fcgr4 and Lamp2), and NETosis marker (Padi4) in mouse lung tissues at day 0, 2, 5, and 7 after SARS-CoV-2 infection. *Red* asterisks indicate the cells in enlarged images. *B–F*, quantification of (*B*) Smg6- (*C*) Upf1-, (*D*) Fcgr4-, (*E*) Lamp2-, and (*F*) Padi4-positive cells per 20X high-power field. Data are shown as mean ± SEM (n = 4 per group). ∗∗, *p* < 0.01; ∗∗∗, *p* < 0.001 by one-way ANOVA with Tukey’s posthoc correction. *G*, representative co-immunofluorescence images for Padi4 (*light blue*) and also for cell type markers (*red*): LAMP3 for type II alveolar epithelial cells, F4/80 for macrophages, and Ly6G/6C for neutrophils at day 2 (*left panel*) and 7 (*right panel*) after SARS-CoV-2 infection.

We next examined which cell types expressed Padi4 by performing immunofluorescence (IF) analysis with type II alveolar epithelial cell marker LAMP3, macrophage marker F4/80, and neutrophil marker Ly6G/6C at day 2 and 7 after SARS-CoV-2 infection. Expression of the NETosis marker Padi4 partly overlapped with those of F4/80 and Ly6G/6C at day 2 (Fig. 4*G*, left panel), indicating its weak expression in macrophages and neutrophils at early stage of infection. In contrast, Padi4 was significantly up-regulated together with Ly6G/6C at day 7, and its expression strongly overlapped with that of Ly6G/6C (Fig. 4*G*, right panel), suggesting that NETosis becomes further aggravated by massive neutrophils at late stage. These data confirm that Padi4 is highly expressed in neutrophils in the late stage of infection, indicating massive activation of NETosis at this stage. Taken together, our temporal proteogenomic analysis suggests three pathway-level markers- NMD (Smg6 and Upf1) for early-to-intermediate, phagocytosis (Fcgr4 and Lamp2)- for intermediate-to-late, and NETosis (Padi4) for late pathological transitions, respectively.

## Discussion

In this study, we presented time-course proteome profiles and analyzed them to elucidate the dynamic activation programs at the protein level. Integrative analysis of (1) early (G1-3), intermediate (G4-6), late (G7-9) upregulated protein groups, (2) cellular pathways enriched by these protein groups, and (3) temporal transitions of the pathways during the course of infection identified NMD, phagocytosis, and NETosis pathways representing early-to-intermediate, intermediate-to-late, and late transitions, respectively. In addition, comparative analysis of our proteome data with a previously reported time-course transcriptome dataset showed that phagocytosis and NETosis-related pathways were consistently up-regulated at intermediate and late stages, respectively, at the mRNA level. Moreover, comparative analyses with (1) human proteome data from autopsy lung tissues and (2) serum proteome data from COVID-19 patients further suggested that NMD, phagocytosis, and/or NETosis-related pathways were predominantly upregulated in patients with severe COVID-19. Taken together, the comparative analyses strongly suggest the association of the three pathways with COVID-19 severity. Finally, the network models proposed biomarkers for the three pathways (Smg6 and Upf1 for NMD, Fcgr4 and Lamp2 for phagocytosis, and Padi4 for NETosis), followed by confirmation of their temporal up-regulation patterns using IHC analysis.

A number of studies have provided pieces of evidence for the association of NMD, phagocytosis, and NETosis with severity in patients with viral infection including COVID-19. NMD degrades not only aberrant host mRNAs, but also targets viral RNAs (e.g., SARS-CoV-2 RNAs with multiple ORFs and long 3′ UTRs (34, 43)). Depletion of core NMD factors (UPF1, UPF2, SMG5, or SMG6) was shown to increase viral titers in mouse hepatitis virus (MHV)-infected fibroblast cells (17Cl-1), demonstrating an inhibitory function of NMD on viral replication (34). In our data, NMD was activated at Day 1 and 2 after infection to inhibit SARS-CoV-2 replication at early stage, but became deactivated after Day 2, as it was enriched by early groups (G1 and 2 including Smg6), showing transient up-regulation at Day 1 and 2. The viral nucleocapsid (N) protein was shown to suppress NMD (35), which could cause the deactivation of NMD, suggesting the presence of replicating SARS-CoV-2 that evaded the initial NMD-mediated viral surveillance. As a compensatory response to inhibit such SARS-CoV-2, NMD was activated again at the intermediate stage, as it was also enriched by an intermediate group (G4), which included UPF1, a central regulator of NMD. The secondary activation of NMD, representing the early-to-intermediate transition thus reflects the failure of the initial NMD-mediated viral surveillance, and thereby replicating SARS-CoV-2 aggravates pro-inflammatory responses, contributing to severity. Taken together, these data suggest that SMG6 and UPF1, representing G1-2 and G6, respectively, can serve as effective biomarkers for predicting COVID-19 severity.

Phagocytosis is a process where antigen presenting cells engulf the infected cells, followed by processing and presentation of viral antigens. Cytotoxic T-cells then recognize the presented viral antigens and kill the infected cells with the antigens. Inhibition of phagocytosis using Cytochalasin D significantly reduced the secretion of pro-inflammatory cytokines (IL6 and TNF) in monocyte-derived macrophages infected with SARS-CoV-2 (44). Phagocytosis is thus strongly activated at intermediate stage to remove the infected cells and virus as well. Consistently, in our data, phagocytosis (mainly Fc gamma receptor-mediated phagocytosis; Fig. 3*C*, top) was activated at intermediate stage, as it was enriched by intermediate groups (G4–6) showing sustained up-regulation up to the late stage (Day 5 and 7). Interestingly, however, phagocytosis was further augmented by the up-regulated phagosome maturation at late stage (Fig. 3*C*, bottom), as it was also enriched by a late group G8. Phagosome maturation has been reported to be antiviral, which can be a compensatory response to replicating SARS-CoV-2 and the infected cells present at late stage. Therefore, the augmentation of phagosome maturation representing the intermediate-to-late transition reflects the presence of replicating SARS-CoV-2 and infected cells that can aggravate pro-inflammatory responses, thereby contributing to severity. We selected the representative biomarkers of FCGR4 and LAMP2 for Fc gamma receptor-mediated phagocytosis and phagosome maturation, respectively. In severe COVID-19 lungs, FCGR3A (a human ortholog of FCGR4)-mediated phagocytosis of SARS-CoV-2 infected cells by monocyte-derived alveolar macrophages (Mo-AMs) triggered IL6 and TNF secretion, inflammasome activation, and pyroptotic cell death, resulting in tissue injury (45). Furthermore, it activated plasmacytoid dendritic cells (pDCs), which in turn secretes high levels of IFN-α and TNF, leading to cytokine amplification (44). LAMP2 binds 5′ UTR of the SARS-CoV-2 RNA, and the absence of the LAMP2A isoform enhanced the viral RNA level, whereas its induction significantly lowered the viral RNA level (46). These data demonstrate pro-inflammatory roles of Fc gamma receptors and antiviral function of LAMP2. Taken together, these data suggest that FCGR4 and LAMP2 representing phagocytosis (intermediate) and phagosome maturation (late), respectively, can serve as effective biomarkers for predicting COVID-19 severity.

NETosis is a process by which neutrophils produce extracellular web-like structure consisting of chromatin components, granule-derived enzymes, and other cytosolic proteins (47). Despite its beneficial role to trap and contain pathogens, when excessively activated, NETosis can be harmful to propagate inflammation, microvascular thrombosis, and tissue/organ damage (48, 49), leading to severe phenotypes. Zuo *et al.* (37) reported that COVID-19 patients showed the elevated serum levels of NETosis-specific markers, myeloperoxidase-DNA (MPO-DNA), and citrullinated histone H3 (Cit-H3), consistent with the increased absolute neutrophil count and platelet levels. Veras *et al.* (38) also showed that the concentration of NETs was increased in plasma, tracheal aspirate, and lung autopsy tissues from severe COVID-19 patients and that the NETs produced by activated neutrophils promoted lung epithelial cell death. In our data, NETosis was strongly activated at late stage, as it was enriched by a late group G8, which included PADI4, a key regulator of both vital and suicidal NETosis. Our network model further showed the late activation of NETosis during the course of infection, and potential associations of key signaling pathways including PI3K, ROS, and inflammasome. In support, these pathways have been proposed as therapeutic targets of severe COVID-19 phenotypes (50, 51, 52). Taken together, these data suggest that PADI4 representing the late excessive activation of NETosis can serve as an effective biomarker for predicting COVID-19 severity.

The abovementioned data demonstrate potential utility of NMD, phagocytosis, and NETosis pathways for predicting COVID-19 severity in clinical settings. Comparative analysis of our proteome data with human lung proteome data showed that NMD, phagocytosis, and NETosis were consistently upregulated in the lungs from patients with severe SARS-CoV-2 infection. Therefore, a panel of biomarkers representing the activation of the three pathways along the course of infection can be designed for prediction of COVID-19 severity for lung biopsy samples. Serum samples are more readily obtained from COVID-19 patients than lung biopsy samples. Comparative analysis with human serum data also showed that phagocytosis and NETosis were consistently up-regulated in serum samples from severe COVID-19 patients. Another panel of biomarkers can be designed for predicting the severity for serum samples because secretion can change the up-regulation pattern of the proteome associated with the pathways. Despite the consistent up-regulation of the selected pathways, due to the difference between mouse and human samples and also between lungs and sera, the validity of the representative markers for the pathways has to be further confirmed using lung biopsy or serum samples from a large cohort of COVID-19 patients. During this validation procedure, alternative representative markers can be selected as more suitable biomarkers because of the distinctive up-regulation patterns in human lung and serum: for example, one of our markers for phagocytosis FCGR4 is only present in mouse, and its human ortholog FCGR3A or other molecules showing much clearer up-regulation patterns in human lung or serum samples can be selected instead. In summary, our time-course proteome analysis provides the basis for dynamic activation of cellular pathways during the course of infection, and key pathways undergoing temporal transitions (early-to-intermediate, intermediate-to-late, and late transitions) as the indicators of severity along the progression of infection.

## Data Availability

All generated proteomics data have been deposited in the ProteomeXchange Consortium (http://proteomecentral.proteomexchange.org) via the PRIDE partner repository, with PXD036193 as the identifier (53, 54).

## Supplemental data

This article contains supplemental data.

## Conflict of interest

The authors declare that they have no conflicts of interest with the contents of this article.

## References

[1] de Bruin S., Bos L.D., van Roon M.A., Tuip-de Boer A.M., Schuurman A.R., Koel-Simmelinck M.J. (2021). Clinical features and prognostic factors in Covid-19: a prospective cohort study. EBioMedicine.

[2] Wu M., Chen Y., Xia H., Wang C., Tan C.Y., Cai X. (2020). Transcriptional and proteomic insights into the host response in fatal COVID-19 cases. Proc. Natl. Acad. Sci. U. S. A..

[3] Nie X., Qian L., Sun R., Huang B., Dong X., Xiao Q. (2021). Multi-organ proteomic landscape of COVID-19 autopsies. Cell.

[4] Winkler E.S., Bailey A.L., Kafai N.M., Nair S., McCune B.T., Yu J. (2020). SARS-CoV-2 infection of human ACE2-transgenic mice causes severe lung inflammation and impaired function. Nat. Immunol..

[5] Ammerman N.C., Beier-Sexton M., Azad A.F. (2008). Growth and maintenance of Vero cell lines. Curr. Protoc. Microbiol..

[6] Park W.B., Kwon N.-J., Choi S.-J., Kang C.K., Choe P.G., Kim J.Y. (2020). Virus isolation from the first patient with SARS-CoV-2 in Korea. J. Korean Med. Sci..

[7] van den Worm S.H., Eriksson K.K., Zevenhoven J.C., Weber F., Züst R., Kuri T. (2012). Reverse genetics of SARS-related coronavirus using vaccinia virus-based recombination. PLoS One.

[8] Ogando N.S., Dalebout T.J., Zevenhoven-Dobbe J.C., Limpens R., van der Meer Y., Caly L. (2020). SARS-coronavirus-2 replication in Vero E6 cells: replication kinetics, rapid adaptation and cytopathology. J. Gen. Virol..

[9] Oladunni F.S., Park J.G., Pino P.A., Gonzalez O., Akhter A., Allué-Guardia A. (2020). Lethality of SARS-CoV-2 infection in K18 human angiotensin-converting enzyme 2 transgenic mice. Nat. Commun..

[10] Han D., Moon S., Kim Y., Kim J., Jin J., Kim Y. (2013). In-depth proteomic analysis of mouse microglia using a combination of FASP and StageTip-based, high pH, reversed-phase fractionation. Proteomics.

[11] Han D., Jin J., Woo J., Min H., Kim Y. (2014). Proteomic analysis of mouse astrocytes and their secretome by a combination of FASP and StageTip-based, high pH, reversed-phase fractionation. Proteomics.

[12] Do M., Kim H., Shin D., Park J., Kim H., Han Y. (2020). Marker identification of the grade of Dysplasia of intraductal papillary mucinous neoplasm in pancreatic cyst fluid by quantitative proteomic profiling. Cancers (Basel).

[13] Kim D.K., Park J., Han D., Yang J., Kim A., Woo J. (2018). Molecular and functional signatures in a novel Alzheimer’s disease mouse model assessed by quantitative proteomics. Mol. Neurodegener.

[14] Kulak N.A., Pichler G., Paron I., Nagaraj N., Mann M. (2014). Minimal, encapsulated proteomic-sample processing applied to copy-number estimation in eukaryotic cells. Nat. Methods.

[15] Kim M.J., Do M., Han D., Son M., Shin D., Yeo I. (2022). Proteomic profiling of postmortem prefrontal cortex tissue of suicide completers. Transl. Psychiatry.

[16] Elias J.E., Gygi S.P. (2007). Target-decoy search strategy for increased confidence in large-scale protein identifications by mass spectrometry. Nat. Methods.

[17] Bolstad B.M., Irizarry R.A., Åstrand M., Speed T.P. (2003). A comparison of normalization methods for high density oligonucleotide array data based on variance and bias. Bioinformatics.

[18] Kim Y., Kim T.K., Kim Y., Yoo J., You S., Lee I. (2011). Principal network analysis: identification of subnetworks representing major dynamics using gene expression data. Bioinformatics.

[19] Bhin J., Jeong H.S., Kim J.S., Shin J.O., Hong K.S., Jung H.S. (2015). PGC-Enriched miRNAs control Germ Cell development. Mol. Cells.

[20] Hwang D., Smith J.J., Leslie D.M., Weston A.D., Rust A.G., Ramsey S. (2005). A data integration methodology for systems biology: experimental verification. Proc. Natl. Acad. Sci. U. S. A..

[21] Huang D.W., Sherman B.T., Lempicki R.A. (2009). Systematic and integrative analysis of large gene lists using DAVID bioinformatics resources. Nat. Protoc..

[22] Ashburner M., Ball C.A., Blake J.A., Botstein D., Butler H., Cherry J.M. (2000). Gene Ontology: tool for the unification of biology. Nat. Genet..

[23] Kanehisa M., Goto S. (2000). KEGG: kyoto encyclopedia of genes and genomes. Nucleic Acids Res..

[24] Hwang D., Schmitt W.A., Stephanopoulos G., Stephanopoulos G. (2002). Determination of minimum sample size and discriminatory expression patterns in microarray data. Bioinformatics.

[25] Schurch N.J., Schofield P., Gierliński M., Cole C., Sherstnev A., Singh V. (2016). How many biological replicates are needed in an RNA-seq experiment and which differential expression tool should you use?. RNA.

[26] Leng L., Cao R., Ma J., Mou D., Zhu Y., Li W. (2020). Pathological features of COVID-19-associated lung injury: a preliminary proteomics report based on clinical samples. Signal. Transduct. Target. Ther..

[27] Suresh V., Mohanty V., Avula K., Ghosh A., Singh B., Reddy R.K. (2021). Quantitative proteomics of hamster lung tissues infected with SARS-CoV-2 reveal host factors having implication in the disease pathogenesis and severity. FASEB J..

[28] Marshall J.S., Warrington R., Watson W., Kim H.L. (2018). An introduction to immunology and immunopathology. Allergy Asthma Clin. Immunol..

[29] Sego T.J., Aponte-Serrano J.O., Ferrari Gianlupi J., Heaps S.R., Breithaupt K., Brusch L. (2020). A modular framework for multiscale, multicellular, spatiotemporal modeling of acute primary viral infection and immune response in epithelial tissues and its application to drug therapy timing and effectiveness. Plos Comput. Biol..

[30] Beristain-Covarrubias N., Perez-Toledo M., Thomas M.R., Henderson I.R., Watson S.P., Cunningham A.F. (2019). Understanding infection-induced thrombosis: lessons learned from animal models. Front. Immunol..

[31] Gindlhuber J., Tomin T., Wiesenhofer F., Zacharias M., Liesinger L., Demichev V. (2022). Proteomic profiling of end-stage COVID-19 lung biopsies. Clin. Proteomics.

[32] Yin S., Klaeger S., Chea V.A., Carulli I.P., Rachimi S., Black K.E. (2023). Integrated Immunopeptidomic and Proteomic Analysis of COVID-19 lung biopsies. Front Immunol..

[33] World Health Organization (2023).

[34] Wada M., Lokugamage K.G., Nakagawa K., Narayanan K., Makino S. (2018). Interplay between coronavirus, a cytoplasmic RNA virus, and nonsense-mediated mRNA decay pathway. Proc. Natl. Acad. Sci. U. S. A..

[35] Nuccetelli V., Mghezzi-Habellah M., Deymier S., Roisin A., Gérard-Baraggia F., Rocchi C. (2025). The SARS-CoV-2 nucleocapsid protein interferes with the full enzymatic activation of UPF1 and its interaction with UPF2. Nucleic Acids Res..

[36] Mukhopadhyay S., Sinha S., Mohapatra S.K. (2021). Analysis of transcriptomic data sets supports the role of IL-6 in NETosis and immunothrombosis in severe COVID-19. BMC Genom Data.

[37] Zuo Y., Yalavarthi S., Shi H., Gockman K., Zuo M., Madison J.A. (2020). Neutrophil extracellular traps in COVID-19. JCI Insight.

[38] Veras F.P., Pontelli M.C., Silva C.M., Toller-Kawahisa J.E., de Lima M., Nascimento D.C. (2020). SARS-CoV-2-triggered neutrophil extracellular traps mediate COVID-19 pathology. J. Exp. Med..

[39] Filbin M.R., Mehta A., Schneider A.M., Kays K.R., Guess J.R., Gentili M. (2021). Longitudinal proteomic analysis of severe COVID-19 reveals survival-associated signatures, tissue-specific cell death, and cell-cell interactions. Cell Rep. Med..

[40] Zhong W., Altay O., Arif M., Edfors F., Doganay L., Mardinoglu A. (2021). Next generation plasma proteome profiling of COVID-19 patients with mild to moderate symptoms. EBioMedicine.

[41] Foo S.S., Cambou M.C., Mok T., Fajardo V.M., Jung K.L., Fuller T. (2021). The systemic inflammatory landscape of COVID-19 in pregnancy: extensive serum proteomic profiling of mother-infant dyads with in utero SARS-CoV-2. Cell Rep. Med..

[42] Byeon S.K., Madugundu A.K., Garapati K., Ramarajan M.G., Saraswat M., Kumar M.P. (2022). Development of a multiomics model for identification of predictive biomarkers for COVID-19 severity: a retrospective cohort study. Lancet Digit Health.

[43] Schmidt N., Lareau C.A., Keshishian H., Ganskih S., Schneider C., Hennig T. (2021). The SARS-CoV-2 RNA–protein interactome in infected human cells. Nat. Microbiol..

[44] García-Nicolás O., Godel A., Zimmer G., Summerfield A. (2023). Macrophage phagocytosis of SARS-CoV-2-infected cells mediates potent plasmacytoid dendritic cell activation. Cell Mol. Immunol..

[45] Junqueira C., Crespo Â., Ranjbar S., de Lacerda L.B., Lewandrowski M., Ingber J. (2022). FcγR-mediated SARS-CoV-2 infection of monocytes activates inflammation. Nature.

[46] Verma R., Saha S., Kumar S., Mani S., Maiti T.K., Surjit M. (2021). RNA-Protein interaction analysis of SARS-CoV-2 5' and 3' untranslated regions reveals a role of lysosome-associated membrane Protein-2a during viral infection. mSystems.

[47] Brinkmann V., Reichard U., Goosmann C., Fauler B., Uhlemann Y., Weiss D.S. (2004). Neutrophil extracellular traps kill bacteria. Science.

[48] Twaddell S.H., Baines K.J., Grainge C., Gibson P.G. (2019). The emerging role of neutrophil extracellular traps in respiratory disease. Chest.

[49] Porto B.N., Stein R.T. (2016). Neutrophil extracellular traps in pulmonary diseases: too much of a good thing?. Front Immunol..

[50] Khezri M.R., Varzandeh R., Ghasemnejad-Berenji M. (2022). The probable role and therapeutic potential of the PI3K/AKT signaling pathway in SARS-CoV-2 induced coagulopathy. Cell Mol. Biol. Lett..

[51] Youn J.Y., Zhang Y., Wu Y., Cannesson M., Cai H. (2021). Therapeutic application of estrogen for COVID-19: attenuation of SARS-CoV-2 spike protein and IL-6 stimulated, ACE2-dependent NOX2 activation, ROS production and MCP-1 upregulation in endothelial cells. Redox Biol..

[52] Zeng J., Xie X., Feng X.L., Xu L., Han J.B., Yu D. (2022). Specific inhibition of the NLRP3 inflammasome suppresses immune overactivation and alleviates COVID-19 like pathology in mice. EBioMedicine.

[53] Vizcaíno J.A., Côté R.G., Csordas A., Dianes J.A., Fabregat A., Foster J.M. (2013). The PRoteomics IDEntifications (PRIDE) database and associated tools: status in 2013. Nucleic Acids Res..

[54] Vizcaíno J.A., Deutsch E.W., Wang R., Csordas A., Reisinger F., Ríos D. (2014). ProteomeXchange provides globally coordinated proteomics data submission and dissemination. Nat. Biotechnol..

