# Supplementary material for: Temporal Proteomic Characterization of SARS-CoV-2-Infected Mouse Lungs
Source: Mol Cell Proteomics. 2026 Jul 2;25(10):101618. doi: 10.1016/j.mcpro.2026.101618 (PMC13697281; doi:10.1016/j.mcpro.2026.101618)
Supplement: Supplementary file 1 [file mmc1.docx]

**Supplemental Figure S1. 10-plex TMT labeling.** The indicated numbers of lung tissue samples from independent mice were first collected from day 0 (normal control), 1, 2, 5 and 7 after SARS-CoV-2 infection. These collected samples were grouped into three sets (Set 1-3) of 10 TMT channels as illustrated in the labeling scheme (Isobaric chemical tag labeling). In this scheme, each sample label indicates the condition and the replicate number (condition_replicate number). In each TMT set, pooled samples were added as universal references (global intermediary) that were used to calculate relative abundances of labeled peptides. Each TMT set was then subjected to desalting and high pH fractionation (**Fig. 1A**). Finally, the resulting fractions were analyzed by LC-MS/MS, and abundances of the peptides were quantified based on the reporter ion intensities in MS/MS spectra.

**Supplemental Figure S2. Identification of early, intermediate, and late elevated proteins along SARS-CoV-2 infection.** (A) 8277 proteins detected in all three TMT sets. The Venn diagram shows 1022 and 840 proteins detected in two TMT sets and only in one TMT set, respectively. There were 705 proteins that were identified, but not quantified by Proteome Discoverer. (B) Numbers of proteins identified in three TMT sets. (C) Protein ranks based on the summed reporter ion intensities of peptides belonging to each detected protein. (D) 43 clusters identified from orthogonal non-negative matrix factorization (ONMF) clustering with 50 bases. Of note, 7 clusters contained no proteins assigned to them and were thus now shown. Colors in the heat maps indicated up- (red) and down-regulation (blue) at day 1, 2, 5, and 7 compared to the control (day 0) after SARS-CoV-2 infection. The clusters were categorized into up- (red box) and down-regulated (blue box) clusters and clusters with the mixed patterns (green box). (E) Merging 21 clusters into early (G1-3), intermediate (G4-6), and late (G7-9) elevated groups. (F) Overlapping up- and down-regulated proteins between 1) the up-regulated proteins in G1-9 from our mouse lung proteome data and 2) the differentially expressed proteins (DEPs; 1259 up-regulated and 347 down-regulated) from the previous proteome of the autopsy lung tissues from the deceased patients with COVID-19. The significance of the overlapping proteins is denoted by -log_10_(q-value) where q-value was obtained by the empirical hypergeometric test followed by the Benjamini-Hochberg correction similar to that described in **EXPERIMENTAL PROCEDURES**. Of note, for this empirical test, a combined background population was generated by taking the union of the 8277 proteins from our data and the 11387 detected proteins in autopsy lung tissues. Two independent random samplings (matching the number of proteins in each of G1-9 and the 1259 up- and 347 down-regulated proteins) were performed from this combined background (11758 proteins), and the empirical null distributions were then estimated for the overlapping proteins between the randomly sampled protein group (G1-9) and the randomly sampled up- or down-regulated proteins (human lung tissues). Dotted line, a cutoff of q = 0.05.

**Supplemental Figure S3. Comparison of the up-regulated proteins and their associated pathways with those from human lung and serum proteomes.** (A) GOBPs or pathways represented by the overlapping proteins between G4-9 and the up-regulated proteins in the previous human autopsy lung proteome. The bars represent −log_10_(q) where q is the enrichment q-value of each GOBP/pathway being enriched by the overlapping proteins, which were calculated by the empirical hypergeometric test method described in **EXPERIMENTAL PROCEDURES** using the reference (background) of 7248 proteins representing the intersection between the autopsy lung proteome and our proteome. Of note, for this enrichment analysis, a related cutoff of 0.1 (dashed line) was used to include important pathways such as neutrophil degranulation and NETosis. See the legend of **Fig. 3A** for the label colors of the terms. (B) 28 clusters identified from orthogonal non-negative matrix factorization (ONMF) clustering of proteins in human serum proteome data with 50 bases. Of note, 22 clusters contained no proteins assigned to them and were thus not shown. For each protein (row), the heat maps showed *t*-statistic values of samples at non-severe, severe, and critical cases (1^st^, 2^nd^, and 3^rd^ columns in the heat map, respectively) versus the control. The clusters were categorized into up- (red box, left) and down-regulated (blue box, middle) clusters and clusters with the mixed patterns (green box, right). (C) Merging the clusters with similar up-regulation patterns into three clusters showing predominant up-regulation at non-severe samples (‘Non-severe’, left), severe and critical samples (‘Severe-critical’, right), and up-regulation at all infected conditions (‘All infected conditions’, middle). For the proteins in each of the three merged clusters, the heat maps in the bottom showed the auto-scaled log_2_(protein abundances) across individual samples (see color bar). (D) Heat map showing GOBPs or pathways represented by the proteins in the three merged clusters. The color bar denotes *Z*-scores computed as −N^−1^(q), where q is the q-value for the pathway from empirical hypergeometric testing using the reference of 1462 human serum proteome, and N^−1^ is the inverse normal distribution. Significant pathways were identified based on the threshold of q < 0.1.

**Supplemental Figure S4. Temporal up-regulation patterns of mRNAs after SARS-CoV-2 infection and their associated pathways.**

(A) Heat maps showing up-regulation patterns of mRNAs in RNA_G1–5 over time. The number of genes in each group is denoted in parentheses. The color bar denotes the gradient of the auto-scaled log_2_(FPKM + 1) for individual mRNAs (row). FPKM, fragments per kilobase of transcript per million. (B) Temporal profiles of the auto-scaled log_2_-fold-changes for individual proteins and log_2_(FPKM + 1) for individual mRNAs (gray lines) in the indicated protein or RNA groups together with the median profiles (black lines). For clear comparison, protein data at day 1 were omitted. The number of molecules in each pattern is denoted in parentheses. (C) Contingency table showing the numbers of overlapping molecules between protein and RNA groups. Background color bar represents the gradient of the significance (q-value) of the overlaps between the corresponding pairs of the protein and RNA groups where q-value was calculated by the empirical hypergeometric test method followed by the Benjamini-Hochberg correction (see the legend in **Supplemental Figure S2F** for random sampling). Pairs of shared patterns shown in (B) were highlighted by blue squares. (D) GOBPs or pathways represented by the mRNAs in RNA_G1–5. Dot color denotes *Z*-scores computed as −N^−1^(q), where q is the enrichment q-value of the pathway, and N^−1^ is the inverse normal distribution, and dot size denotes the number of mRNAs involved in the corresponding GOBPs or pathways. Colored boxes next to each term indicate whether the term is also enriched by the up-regulated proteins (Protein_G1–9 in **Fig. 2B**) with the same (dark blue) or different (light blue) kinetic patterns. White boxes indicate no enrichment by the protein groups. See the legend of **Fig. 3A** for the label colors of the terms. (E) Classes 1–4 of the pathways categorized by whether they were enriched by the mRNAs and/or proteins with the shared or distinctive kinetic patterns. See text for definition of Classes 1–4. (F) Heat maps showing expression patterns of proteins and mRNAs involved in the indicated GOBPs or pathways. For Phagocytosis and NETosis, expression patterns of proteins and mRNAs from the indicated protein and RNA groups were shown. For NMD, proteins were selected from Protein_G1, G2, and G4 that significantly represented NMD, and expression patterns of these selected proteins and their corresponding mRNAs were shown. For PI3K-Akt signaling pathway, oppositely, mRNAs were selected from RNA_G5 that significantly represented PI3K-Akt pathway, and expression patterns of these selected mRNAs and their corresponding proteins were shown. Colors represent up-regulation (red) and down-regulation (blue) of proteins at day 1, 2, 5, and 7 or mRNAs at day 2, 4, and 7 compared to the control (day 0) after SARS-CoV-2 infection. The color bar denotes the gradient of *t*-values between day 0 and day 1, 2, 5, or 7.

**Supplemental Tables**

**Supplemental Table S1. List of protein-protein interactions and references**

| **Protein-Protein Interaction** | **Network Model** | **Reference** |
| --- | --- | --- |
| immunity-related GTPases | Fig. 1D | Hunn et al., Mamm Genome 22, 43–54 (2011). |
| IRGs (Irga6) disrupts viral replication complex | Fig. 1D | Biering et al., Cell Host Microbe 22, 74–85 (2017) |
| Gbps disrupts viral replication complex | Fig. 1D | Biering et al., Cell Host Microbe 22, 74–85 (2017) |
| Gbp2/5 interferes spike protein cleavage | Fig. 1D | Mesner et al., PNAS 120, e2212577120 (2023) |
| Gbp7 binds Cybb and mediates Nox2 assembly | Fig. 1D | Tretina et al., J Exp Med 216, 482–500 (2019) |
| Rsad2 (viperin) perturbs HCV viral replication | Fig. 1D | Helbig et al., Hepatology 54, 1506–1517 (2011) |
| Zc3hav1 and its cofactor Trim25 inhibits viral RNA replication and translation | Fig. 1D | Nchioua et al., mBio 11, 10–1128 (2020) |
| Isg20 degrades viral RNA and inhibits viral RNA translation | Fig. 1D | Deymier et al., FEBS Open Bio 12, 1096–1111 (2022) |
| Rigi recognizes viral RNA | Fig. 1D | RIG-I-like receptor signaling pathway - Mus musculus (house mouse; KEGG pathway) |
| Oasl2 binds to RIG-I to enhance its signaling activity | Fig. 1D | Zhu et al., Immunity 40, 936–48 (2014) |
| Ifit3 induces Ifit1/2 and they Sting1 and Mavs pathway | Fig. 1D | Chikhalya et al., Mbio 12, (2021) |
| Unc93b1 trafficks Tlr3 and Tlr7 to endosomal compartments, and further stabilizies them | Fig. 1D | Kim et al., Nature 453, 234–238 (2008) / Pelka et al., Immunity 48, 911–922 (2018) |
| Cd40 signaling activates NFkB through noncanonical pathway | Fig. 1D | NF-kappa B signaling pathway - Mus musculus (house mouse; KEGG pathway) |
| Tlr3/7 recognizes viral RNA and Tlr3 activates Traf3 | Fig. 1D | Coronavirus disease - COVID-19 - Mus musculus (house mouse; KEGG pathway) |
| Oasl1 binds to 5'-UTR of Irf7 to inhibit its translation | Fig. 1D | Zhu et al., Immunity 40, 936–48 (2014) |
| Trim21 activates Nmi-Ifi35 complex which further inhibits Irf7 | Fig. 1D | Das et al., Virology 485, 383–392 (2015) |
| Daxx, Pml, Sp100 forms ND10 complex | Fig. 1D | Herpes simplex virus 1 infection - Mus musculus (house mouse; KEGG pathway) |
| Sp110 localizes to the PML-Sp100 nuclear bodies | Fig. 1D | Bloch et al., Mol Cell Biol 20, 6138–6146 (2000) |
| MHC I antigen processing and presentation pathway | Fig. 1E | Antigen processing and presentation - Mus musculus (house mouse; KEGG pathway) |
| Complement and coagulation cascades | Fig. 1F | Complement and coagulation cascades - Mus musculus (house mouse; KEGG pathway) |
| SMG1 phosphorylates UPF1, which in turn recruits SMG6, an endonuclease | Fig. 3B | Kurosaki et al., Nat Rev Mol Cell Biol 20, 406–420 (2019) |
| Phagosome maturation | Fig. 3C | Phagosome - Mus musculus (house mouse; KEGG pathway) |
| Complement-mediated phagocytosis | Fig. 3C | Uribe-Querol and Rosales, Front Immunol 11, 1066 (2020) |
| Fc gamma R-mediated phagocytosis | Fig. 3C | Fc gamma R-mediated phagocytosis - Mus musculus (house mouse; KEGG pathway) |
| Neutrophil extracellular trap formation | Fig. 3D | Neutrophil extracellular trap formation - Mus musculus (house mouse; KEGG pathway) |

**Supplemental Table S2. List of identified peptides.** See Supplemental Table 2.xlsx. Annotated Sequence, the identified peptide sequence with the flanking residues from the master protein; Master Protein Accessions, accessions of all master proteins from all protein groups that include the given peptide sequence; Positions in Master Proteins, positions of the peptide in the master protein; Modifications, all the PTMs identified for the given peptide; Contaminant, true if the proteins were marked as contaminants in the searched FASTA file; Qvality PEP (posterior error probability), the posterior error probability (PEP) for the identified peptide group; Qvality q-value, the q-value for the identified peptide group; # Protein Groups, the number of protein groups that include the peptide; # Proteins, the number of proteins that include the PSM; # PSMs, the total number of identified PSMs in the peptide group; # Missed Cleavages, the number of cleavage sites in a peptide sequence that a cleavage reagent (enzyme) did not cleave; and Theo. MH+ [Da], the mass calculated from the sequence.

**Supplemental Table S3. List of identified proteins.** See Supplemental Table 3.xlsx. Protein FDR confidence, the level of confidence for the identified protein as determined by the Protein FDR Validator node; Accession, the UniProt identifier assigned to the protein by the FASTA database; Protein descriptions; Groups, groups to which each protein was assigned by the clustering analysis; Exp. Q-value, the q-values derived from the validation; Sum PEP Score, the scores that the Protein FDR Validator node calculates on the basis of the PEP values of the PSMs; Coverage [%], the percentage of the protein sequences covered by identified peptides; # Peptides, the total number of distinct peptide sequences identified from all included searches; # PSMs, the number of identified peptide spectrum matches identified from all included searches, including those redundantly identified; # Unique Peptides, the total number of distinct peptide sequences unique to the protein group; and # Protein Groups: the total number of protein groups; # AAs, the length of the protein sequence; MW [kDa]: the calculated molecular weight of the protein; and Abundances, the normalized relative abundance values of proteins in individual samples with respect to those in the universal reference samples.

**Supplemental Table S4. List of mRNAs from the GSE154104 dataset.** See Supplemental Table 4.xlsx. Ensembl gene ID; Entrez gene ID; Gene symbol; Gene description; Groups: groups to which each mRNA was assigned by the clustering analysis; and Abundances, Expression levels in fragments per kilobase of transcript per million mapped reads (FPKM).
